# No Association Between Radiation Dose and Clinical Outcomes in Merkel Cell Carcinoma in the Veteran Population

**DOI:** 10.1016/j.adro.2026.102029

**Published:** 2026-03-17

**Authors:** Sudarshan Karki, Cymon N. Kersch, Ryan A. Melson, Reid F. Thompson

**Affiliations:** a Portland VA Research Foundation, Portland, Oregon; b Department of Radiation Medicine, Oregon Health and Science University, Portland, Oregon; c VA Portland Healthcare System, Portland, Oregon

## Abstract

**Purpose:**

Merkel cell carcinoma (MCC) is a rare, aggressive skin cancer. Treatment often includes high-dose radiation therapy (RT); however, studies suggest that even 8 Gy in 1 fraction can provide local control.

**Methods and Materials:**

In this retrospective, IRB-approved study, we mined the national Veterans Affairs Corporate Data Warehouse for MCC patients treated with RT. Using a combination of manual chart review and semiautomated data extraction from free-text notes, we collected information on diagnosis, tumor site and size, treatment technique, and radiation dose/fractionation. We assessed local and distant tumor control and overall survival as a function of radiation dose.

**Results:**

We identified 324 patients with 618 treated sites that were evaluable. Of these, 386 sites were treated for microscopic disease and 232 for gross disease. Among the gross disease sites, 64 were treated with curative intent, 164 with palliative intent, and 4 with neoadjuvant intent. The 5-year local progression-free probability (LPFP) was 94.0% across the entire cohort, with 1-, 3-, and 5-year LPFP of 92%, 89%, and 86%, respectively, for patients with gross disease. Local tumor control was excellent across the entire spectrum of delivered radiation doses, ranging from 7.5 to 122.4 Gy (biologically effective dose, α/β = 10), both for sites with gross disease and those treated for microscopic disease risk. Local tumor control was 90% to 95% in both the highest and lowest RT dose groups. Univariate and multivariate Cox proportional hazard assessment of radiation dose showed no significant association with LPFP (hazard ratio, 0.99; 95% confidence intervals [CIs], 0.97-1.01; *P* = .22 and hazard ratio, 1.00; 95% CI, 0.97-1.02; *P* = .67, respectively). Distant progression-free probability was also independent of radiation dose.

**Conclusions:**

MCC is generally sensitive to RT, with traditionally palliative doses demonstrating similar local and distant disease control compared with higher dose regimens. Acknowledging several caveats to this work, these data support considering dose de-escalation as a treatment option for MCC.

## Introduction

Merkel cell carcinoma (MCC) is an aggressive, cutaneous, neuroendocrine malignancy, with 5-year survival rates of 62.8% for stage I, 34.8% to 54.6% for stage II, 26.8% to 40.3% for stage III, and 13.5% for stage IV.^1^ Although rare, with 2488 cases per year in the United States in 2013, the incidence of MCC has been increasing over the past 20 years.^2^^,^^3^ MCC was initially thought to arise from Merkel cells, which are present in the basal layer of the skin, where they function as mechanoreceptors for light touch and are associated with afferent nerves and neuroendocrine features.^2^^,^^4^ However, recent studies suggest that transformed fibroblasts or other stem cells may instead be the primary cell type of origin.^5^ Their malignant transformation can be caused by chronic ultraviolet light exposure^6^ and/or integration of the Merkel cell polyomavirus,^7^ which is present in up to 80% of cases in the United States.^8^ Additional risk factors associated with MCC include older age, male sex, light skin, and an immunocompromised state.^1^

Although clinical practice may vary, most patients with localized MCC are treated according to the National Comprehensive Cancer Network (NCCN) guidelines, with primary surgical resection and sentinel lymph node biopsy, often followed by adjuvant dose radiation therapy (RT). For various inoperable cases, RT may be used alone or in combination with systemic therapy, with an estimated in-field tumor control rate of approximately 70% to 95%.^9^, ^10^, ^11^ However, NCCN-recommended adjuvant (50-60 gray [Gy]) and definitive RT (60-66 Gy) doses are often associated with significant toxicities, including but not limited to acute radiation dermatitis with desquamation and risk of bleeding, mucositis, pain, chronic skin changes/fibrosis, lymphedema, and long-term inhibition of local wound healing.^12^, ^13^, ^14^

On the other hand, palliative intent doses of RT (eg, 30 Gy in 10 fractions, 20 Gy in 4 to 5 fractions, or 8 Gy in 1 fraction) may be used for local treatment of advanced MCC with the expectation of reduced radiation treatment–related toxicity and a shorter treatment course.^15^ Although lower RT doses typically reduce treatment response rates, both in vitro^16^ and clinical data^13^^,^^17^^,^^18^ suggest that MCC can be sensitive to RT at these lower doses. For instance, 8 Gy single fraction RT to MCC metastases resulted in durable in-field control with no local progression in 77% (69/89) of treated sites across 16 patients who survived across the study duration, with a median follow-up of 8.4 months.^18^ Even in the adjuvant setting of resected localized MCC of the head and neck, 8 Gy single fraction RT resulted in 100% local control rate among 12 patients with no toxicities greater than grade 1 (per the National Cancer Institute Common Terminology Criteria for Adverse Events system).^17^ An additional study demonstrated comparable in-field local recurrence and disease-specific survival for MCC treated with hypofractionated RT (>2 Gy per fraction) compared to standard fractionated RT, albeit often to a higher total biologically effective dose (BED) than typical palliative doses, suggesting that prolonged courses of RT may be unnecessary for treating MCC.^11^

The promising outcomes observed with lower, palliative-level RT doses for advanced MCC highlight the potential for dose de-escalation in the adjuvant setting where RT is intended to treat microscopic disease. Exploring this question is challenging as many studies in this space are confined to small single-institution cohorts due to the rarity of MCC. Thus, we sought to evaluate long-term control of MCC with RT (in-field and distant disease control) as a function of RT dose in a nationwide cohort of veterans with MCC.

## Methods and Materials

### Cohort construction

**Figure 1:**
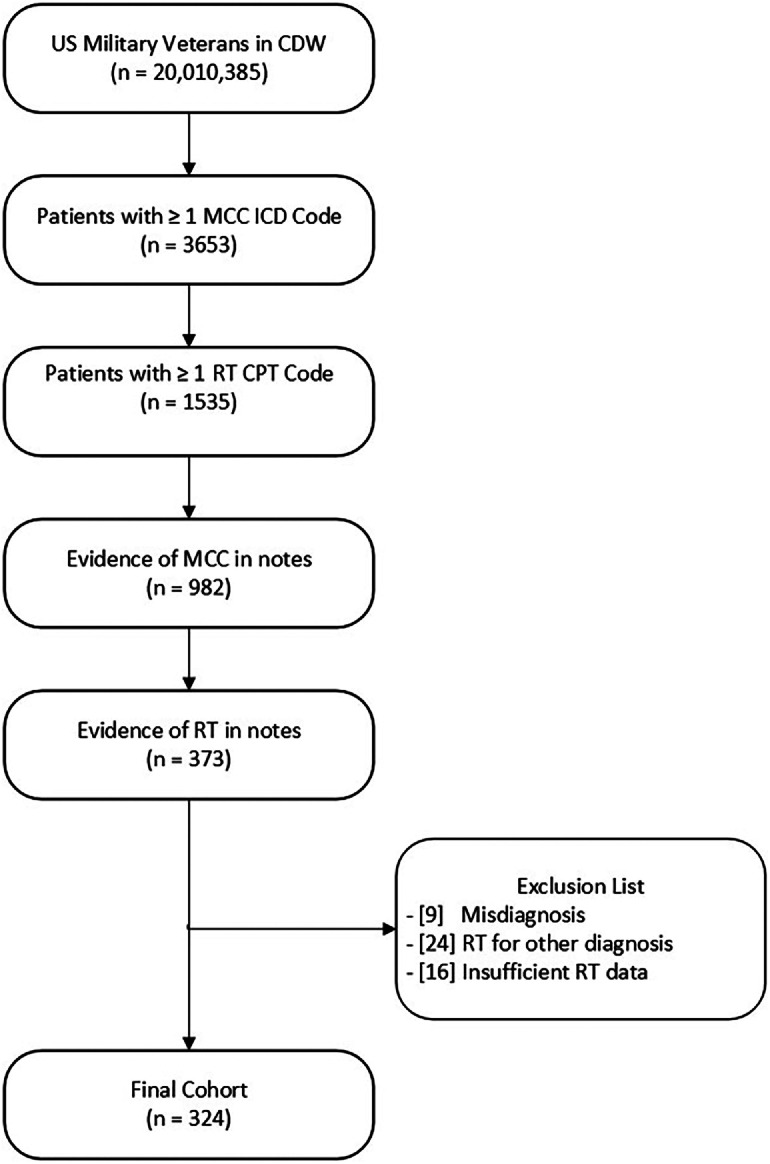
Flowchart showing the study’s cohort selection criteria. *Abbreviations:* CDW = Corporate Data Warehouse; CPT = Current Procedural Terminology; ICD = International Classification of Diseases; MCC = Merkel cell carcinoma; RT = radiation therapy; US = United States.

All work on this study was conducted under Veterans Affairs (VA) Portland Health Care System IRB #4426. We first queried the VA Corporate Data Warehouse to identify all veterans with at least one diagnosis of MCC per International Classification of Diseases (ICD)-9 (209.3X, 209.75) or ICD-10 codes (C4A.X, C7B.1X). From this population, we identified patients with one or more current procedural terminology procedure code(s) for RT (Table E1) occurring on or after the date of their MCC diagnosis. To improve fidelity of both MCC diagnosis and RT treatment status, we further restricted the cohort based on the presence of the following terms in one or more clinical notes: “MCC” or “Merkel” for MCC diagnosis; and “Gy,” “cGy” or “centigray” for RT treatment. To confirm MCC diagnosis and RT treatment, as well as extract meaningful clinical covariates and endpoints, we performed in-depth manual chart review for 373 patients resulting from our cohort identification methodology as above (Fig. 1). We excluded a subset of these patients from our final cohort due to several issues identified during manual chart review, including misdiagnosis, RT treatment for an alternate diagnosis, and insufficient confirmatory records (Fig. 1). The finalized and validated cohort consisted of 324 patients with detailed diagnosis, treatment, and clinical outcome information.

### Covariates and endpoints

We extracted the following 20 features and outcome variables for the full cohort on a site-specific basis, resulting in 618 unique treated sites:

1) Disease site: The anatomic location of each treated site, as documented in clinical notes, was grouped into the following body regions: head/neck, torso, upper extremities, and lower extremities. Axillary sites were considered part of the upper extremities, supraclavicular sites were grouped with the head/neck region, and hip sites were considered part of the lower extremity. Additionally, each treated site was classified as primary tumor, lymph node, in-transit disease, or distant metastasis.
2) Stage: The pathologic and/or clinical stages were extracted directly from clinical notes where present, and were otherwise inferred from the overall clinical record based on available evidence. In cases where pathologic and clinical stages disagreed, we assigned the overall stage based on the more advanced assessment of the 2. Due to the lack of sufficient tumor size measurements among many patients, we were unable to reliably differentiate stage I from stage II disease. For analytical purposes, the stage was ultimately considered as localized (stage I-II), regional (stage III), or metastatic (stage IV).
3) RT dose: Total dose (measured in Gy) delivered to the treated site in a single treatment course. In cases where the dose was heterogeneously prescribed (eg, dose-painting IMRT), the RT dose reflected the highest delivered dose prescription encompassing the treated site in question. Note that VA practice patterns and RT dose utilization could be variable across the enterprise (ie, there was no explicit RT dose expectation applied in this clinical cohort).
4) RT fractionation: Number of RT fractions (individual treatment sessions) administered in a single treatment course for each treated site.
5) BED_10_ (Biologically Effective Dose, α/β ratio of 10): The RT dose and fractionation were used to calculate the BED_10_ using an α/β ratio of 10 and ignoring cellular repopulation.^19^ BED_10_ was included as a continuous variable to capture the continuum of radiation dose, controlling for differing dose-fractionation schemes.
6) RT timeframe: Date of first and last radiation fractions for each treated site. These data were not directly included in any subsequent modeling; however, they were used to determine pre- and post-RT patient timelines, including assessment of time-to-event endpoints.
7) RT modality: Treatment with electrons, photons, or a combination of both, according to the chart review.
8) Treatment intent: Categorized as neoadjuvant, definitive, palliative, or adjuvant/prophylactic for each site, based on detailed review of treatment plans and clinical notes. RT to lymph node site(s) without clinical or pathologic evidence of nodal disease was categorized as prophylactic treatment, whether or not surgery was performed. However, RT to any operated site (eg, via primary excision, sentinel lymph node biopsy, or nodal dissection) having clinical or pathologic evidence of disease was categorized as adjuvant treatment, unless there was recurrent or residual gross disease at the time of initiation of RT, in which case it was instead considered as definitive or palliative treatment according to the broader clinical context. Given the small number of prophylactic cases and the assumption that their underlying risk profile is similar to patients with positive but surgically cleared nodes, we combined the prophylactic and adjuvant groups for statistical analysis. Treatment of gross disease in the absence of prior surgery to that site was considered as neoadjuvant, definitive, or palliative according to the broader clinical context.
9) Systemic therapy: Categorized as either chemotherapy or immunotherapy depending on the agents(s) administered between the start of RT and the date of response assessment. Cases with confirmed absence of systemic treatment during the period were classified as “None,” while those with no or inconclusive information were categorized as “Unknown.”
10) Surgery: If performed, both the date of surgery and the corresponding margin status (categorized as positive, negative, or unknown) were recorded for each tumor.
11) Site-specific disease burden: Categorized as either microscopic (eg, for adjuvant or prophylactic RT) or gross disease (eg, for palliative or definitive RT).
12) Tumor size: For gross disease, tumor size (and corresponding date) was assessed for the nearest pretreatment and all subsequent (posttreatment) timepoints based on available imaging reports, surgical notes, pathology reports, and other clinical notes. Tumor size was estimated as volume (cubic centimeter [cc]) calculated using the ellipsoid formula from available dimension measurements; in cases with fewer than 3 available dimension measurements, the volume was approximated conservatively using the smallest value among existing dimension(s) as the value for missing dimension(s). In cases of microscopic disease (ie, for adjuvant or prophylactic RT) or clinical complete responses, the tumor size was assigned a 0 value.
13) Tumor type: Each tumor was classified as primary (site of initial cutaneous occurrence of MCC), in-transit (cutaneous in-transit recurrence), lymph node (involvement of regional nodal basins), or metastatic (distant site).
14) Age at diagnosis: Calculated as the time difference (in years) between the first appearance of an MCC diagnosis code and the patient’s date of birth.
15) Patient sex: Male or female, according to available demographic records.
16) Patient comorbidity burden: Assessed using the Charlson comorbidity index,^20^ as established in prior work,^21^ to account for the impact of concurrent comorbid health conditions at the time of RT initiation.
17) Immune status: Each patient’s immune state at the time of RT was assessed as a binary yes/no (immunocompromised vs immune intact) based on the presence or absence of a preceding solid organ transplant, clinical documentation of immunosuppression around the time of MCC treatment, or a diagnosis of chronic lymphocytic leukemia or small lymphocytic lymphoma.
18) Local progression: This was our primary outcome and was determined as the first date of disease recurrence or measurable progression (>20% increase in size from the baseline at the start of RT) at the treated site, based on clinical documentation and imaging reports. Note that detailed radiation dose maps were unavailable for review at the time of this study.
19) Distant progression: This was our secondary outcome and was determined for each treated site as the first date of appearance of new lesion(s) outside of the presumptive radiation treatment field (assumed to extend ∼5 cm from the treated site) or, in rare cases of distant progression while on treatment, the date of radiation completion. As noted above, this determination was based primarily on clinical notes and imaging reports documented in the medical record; detailed radiation dose maps were unavailable for review at the time of this study.
20) Overall survival (OS): This was our tertiary outcome and was determined for each treated site as the time from start of radiation treatment to date of death (where relevant).t

### Statistical analysis

Descriptive statistics were used to summarize the baseline characteristics of the patient cohort. Continuous variables were expressed as median (interquartile range), while categorical variables were presented as frequencies and percentages.

The Kaplan-Meier method,^22^ implemented via the lifelines (v 0.28.0) package^23^ in Python (v3.10), was used to estimate the local recurrence and local progression-free probability (LPFP) and distant progression-free probability (DPFP). Survival curves were generated on a per-treated-site basis, and the time to both local and distant progression was calculated for each site as the time between the end of radiation treatment to that site and the first documented local and distant progression, respectively. Survival rates were calculated and reported at clinically relevant time points following RT. The differences between groups were assessed using the log-rank test, with a *P* value <.05 considered statistically significant, adjusting for multihypothesis testing where relevant by the Bonferroni method.

For cases where gross disease was treated with radiation, the tumor’s response was evaluated by comparing its size (approximated by volume) before and after treatment. The measured posttreatment tumor volumes were normalized to the pretreatment volume by calculating a ratio (posttreatment volume divided by pretreatment volume). To support consistent comparison across patients with varying baseline tumor sizes, we assessed relative posttreatment tumor volumes as proportions of corresponding pretreatment volumes. In cases where only standardized uptake values (SUV) were available, response was normalized to the anticipated background SUV level of 2.5 as follows: (posttreatment SUV uptake – 2.5) divided by (pretreatment SUV uptake – 2.5). These normalized values were tracked over time, and tumor responses were classified into 4 categories: progression (>20% interval tumor growth), partial response (>30% sustained reduction in tumor volume or avidity), stable (minimal to no reduction or growth), and complete response (tumor disappearance).

To elucidate the role of radiation dose on local tumor control, we first categorized delivered RT dose according to BED_10_ ranges calculated from current NCCN guidelines and standard palliative dose fractionation, resulting in 6 BED_10_ dose ranges: (1) ≤28 Gy, (2) >28 Gy and ≤39 Gy, (3) >39 Gy and ≤55.2 Gy, (4) >55.2 Gy and ≤60 Gy, (5) >60 Gy and <72 Gy, and (6) ≥72 Gy. We calculated the percentage of tumors with local progression for each subset of sites treated with RT according to a given dose group. Additionally, to capture more fine-grain dose-response relationships, we estimated local control rates at every available BED_10_ data point.

To assess the impact of various covariates on LPFP and DPFP, we constructed both univariate and multivariate Cox proportional hazards regression models and reported hazard ratios (HR) with 95% confidence intervals (CIs). In our analysis, we comprehensively included all clinical factors known or suspected to influence outcomes. *P* values for univariate Cox models were adjusted for 18 comparisons by Bonferroni correction,^24^ resulting in a corrected alpha threshold of 0.0028.

## Results

### Patient cohort

We evaluated 324 US veterans who were treated with RT for their confirmed MCC diagnosis (Table 1). The cohort had a median age of 74 years at the time of MCC diagnosis, and the overwhelming majority of patients were male (98.8%). These patients had a combined total of 618 unique sites treated with RT, with the highest percentage of sites located in the head and neck region (n = 333; 53.9%). RT was targeted primarily to primary sites and lymph nodes (n = 515; 83.3%), with fewer cases targeting in-transit or distant metastatic sites (n = 103;16.7%). Most patients presented with advanced disease (stage III: 47.9% [n = 296], stage IV: 24.6% [n = 152]) at the time of treatment. Surgery was performed prior to RT in 62.1% (n = 381) of cases, with negative surgical margins reported in nearly half of those cases (n = 180; 47.2%). These procedures included excisions of primary cutaneous tumors, nodal excisions/dissections, and metastatectomies. The majority of sites were treated with a dose >55.2 Gy BED_10_ RT in accordance with NCCN guidelines (n = 447; 72.33%). Systemic therapy was used in only a minority of cases (n = 165; 26.7%), with 109 cases (60%) receiving chemotherapy and 66 cases (40%) receiving immunotherapy. Clinically documented immunosuppression during the time of treatment was uncommon (n = 80; 12.9%). Median follow-up varied by cohort (eg, 28.19 months for microscopic disease risk vs 7.95 months for gross disease).

**Table 1:** Characteristics of VA patients and their MCC tumors treated with radiation therapy

Cohort characteristics (n = 324)					
Age at diagnosis, years		74 (69-81)			
Sex	M	320 (98.8%)			
	F	4 (1.2%)			
Tumor characteristics (n = 618)					
		Microscopic risk (n = 386, 62.5%)	Gross disease (n = 232, 37.5%)	Total (n = 618)	*P* value
Treated site	Head and neck	233 (60.4%)	100 (43.1%)	333 (53.9%)	<.001
	Upper extremity	81 (21.0%)	31 (13.4%)	112 (18.1%)	.018
	Torso	31 (8.0%)	71 (30.6%)	102 (16.5%)	<.001
	Lower extremity	41 (10.6%)	30 (12.9%)	71 (11.5%)	.435
Treated site type	Primary	200 (51.8%)	41 (17.7%)	241 (39.0%)	<.001
	Lymph node	172 (44.6%)	102 (44.0%)	274 (44.3%)	.933
	In-transit	10 (2.6%)	30 (12.9%)	40 (6.5%)	<.001
	Metastasis	4 (1.0%)	59 (25.4%)	63 (10.2%)	<.001
Clinical stage	I-II	135 (35.0%)	15 (6.5%)	150 (24.3%)	<.001
	III	208 (53.9%)	88 (37.9%)	296 (47.9%)	<.001
	IV	35 (9.1%)	117 (50.4%)	152 (24.6%)	<.001
	Unknown	8 (2.1%)	12 (5.2%)	20 (3.2%)	.057
RT intent	Neoadjuvant	0 (0.0%)	4 (1.7%)	4 (0.6%)	.020
	Adjuvant/prophylactic	386 (100.0%)	0 (0.0%)	386 (62.5%)	<.001
	Definitive	0 (0.0%)	64 (27.6%)	64 (10.4%)	<.001
	Palliative	0 (0.0%)	164 (70.7%)	164 (26.5%)	<.001
RT modality	Photons	177 (45.9%)	114 (49.1%)	291 (47.1%)	.454
	Electrons	65 (16.8%)	41 (17.7%)	106 (17.2%)	.826
	Mixed	17 (4.4%)	8 (3.4%)	25 (4.0%)	.675
	Unknown	127 (32.9%)	69 (29.7%)	196 (31.7%)	.423
Surgery	Yes	366 (94.8%)	15 (6.5%)	381 (62.1%)	<.001
	No	9 (2.3%)	212 (91.4%)	221 (35.1%)	<.001
	Unknown	11 (2.9%)	5 (2.2%)	16 (2.8%)	.795
Margin	Negative	180 (46.6%)	0 (0.0%)	180 (29.1%)	<.001
	Positive	39 (10.1%)	8 (3.4%)	47 (7.6%)	.003
	No Surgery	9 (2.3%)	212 (91.4%)	221 (35.8%)	<.001
	Unknown	158 (40.9%)	12 (5.2%)	170 (27.5%)	<.001
Systemic therapy	Chemotherapy	45 (11.7%)	64 (27.6%)	109 (17.6%)	<.001
	Immunotherapy	17 (4.4%)	49 (21.1%)	66 (10.7%)	<.001
	None	282 (73.1%)	101 (43.5%)	383 (62.0%)	<.001
	Unknown	42 (10.9%)	18 (7.8%)	60 (9.7%)	.261
Immunosuppression	Yes	38 (9.8%)	42 (18.1%)	80 (12.9%)	.004
	No	311 (80.6%)	167 (72.0%)	478 (77.3%)	.017
	Unknown	37 (9.6%)	23 (9.9%)	60 (9.7%)	.889
RT dose (BED_10_)	≤ 28 Gy	9 (2.3%)	50 (21.6%)	59 (9.5%)	<.001
	>28 Gy, ≤39 Gy	6 (1.6%)	49 (21.1%)	55 (8.9%)	<.001
	>39 Gy, ≤55.2 Gy	29 (7.5%)	28 (12.1%)	57 (9.2%)	.063
	>55.2Gy, ≤60 Gy	129 (33.4%)	28 (12.1%)	157 (25.4%)	<.001
	>60 Gy, <72 Gy	96 (24.9%)	25 (10.8%)	121 (19.6%)	<.001
	≥72 Gy	117 (30.3%)	52 (22.4%)	169 (27.3%)	.040
Follow-up months, median (IQR)	≤ 28 Gy	34.37 (10.09-37.45)	3.75 (1.39-12.47)	4.80 (1.53-15.70)	.042
	>28 Gy, ≤39 Gy	25.95 (15.39-43.17)	4.63 (1.81-7.98)	6.18 (2.10-11.43)	.001
	>39 Gy, ≤55.2 Gy	24.48 (13.54-72.87)	8.49 (6.14-30.88)	17.12 (7.95-37.36)	.002
	>55.2Gy, ≤60 Gy	41.40 (17.05-75.30)	9.30 (4.72-42.73)	32.92 (11.56-68.67)	<.001
	>60 Gy, <72 Gy	23.89 (11.52-46.75)	13.34 (5.59-37.26)	20.76 (8.80-42.91)	.120
	≥72 Gy	27.70 (12.48-66.83)	23.15 (7.61-42.31)	26.74 (10.91-62.78)	.086
	Overall	28.19 (12.57-65.15)	7.95 (3.21-23.65)	26.83 (7.36-51.88)	<.001

*Abbreviations:* IQR = interquartile range; MCC = Merkel cell carcinoma; RT = radiation therapy.

### Local tumor control

**Figure 2:**
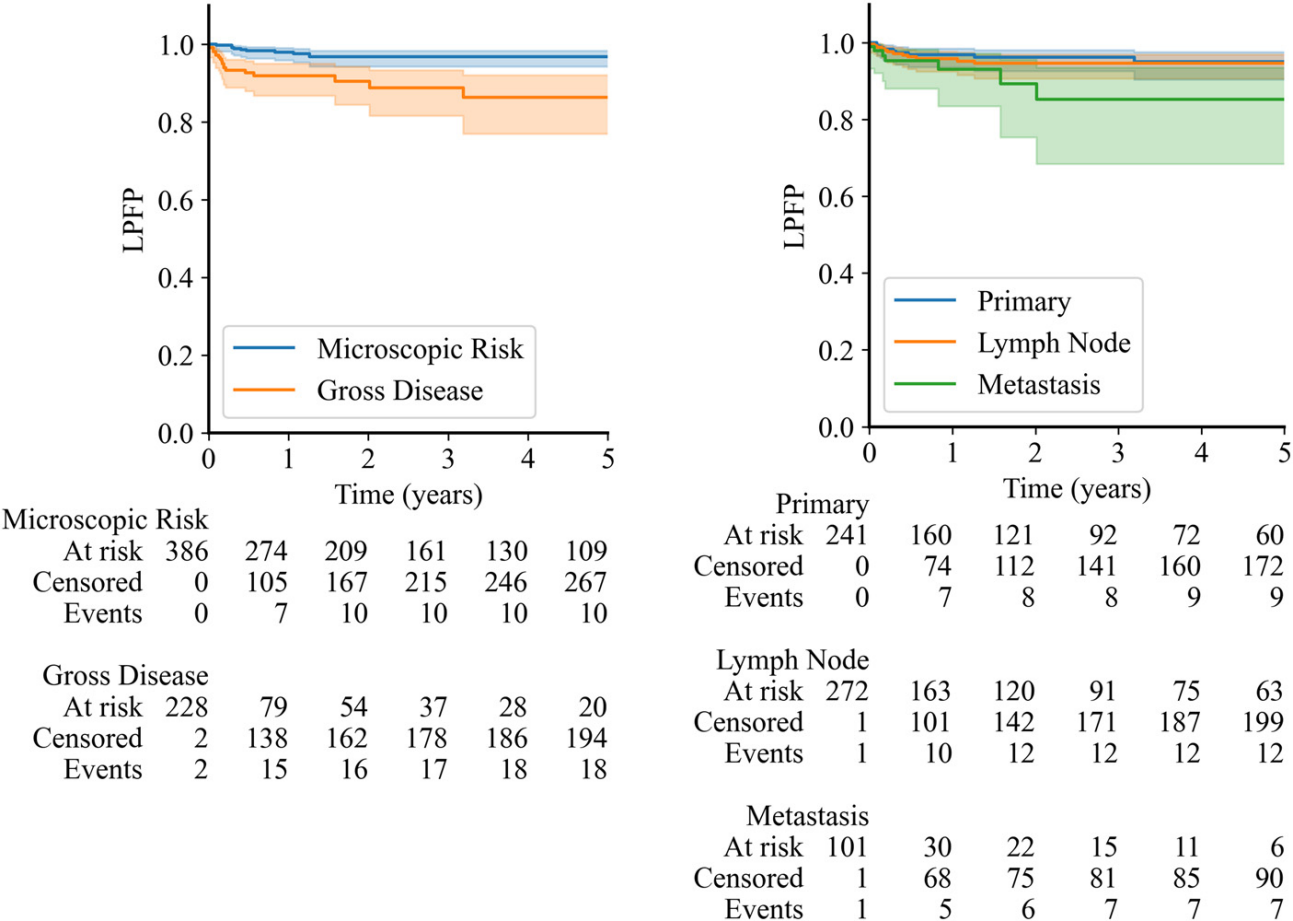
Kaplan-Meier curves showing local progression-free probability (LPFP). (LEFT) LPFP of sites treated for either microscopic disease risk after surgical resection (blue line) or macroscopic gross disease (includes both primary and distant metastatic sites) (orange line) (*P* < .0001). (RIGHT) LPFP at primary tumor sites (blue line), lymph nodes (orange line), and secondary/metastatic tumor sites (green line) after radiation therapy. No pairwise comparisons between groups reached statistical significance.

Our primary objective was to assess the efficacy of RT on local MCC tumor control. Local control was excellent, with a 3-year LPFP of 94.0% across the entire study cohort (Fig. E1). As local tumor control rates are often lower in patients treated with gross disease versus those treated for microscopic disease risk, we compared LPFP between these subgroups (Fig. 2A). Although local control was indeed higher in the microscopic risk versus gross disease setting (*P* < .0001), LPFP remained excellent in the gross disease setting (1-year LPFP of 92%). When further stratified by treatment site, local control appeared slightly better for primary sites and lymph nodes compared to metastatic sites, although these differences did not reach statistical significance (Fig. 2B). Among metastatic sites, LPFP outcomes were comparable between in-transit and distant metastasis, though interpretation is limited by small number of treated sites in each group (Fig. E2A, *P* = .57). Local control rates remained similar across clinical stage at time of treatment in both microscopic and gross disease settings (Fig. E3).

**Figure 3:**
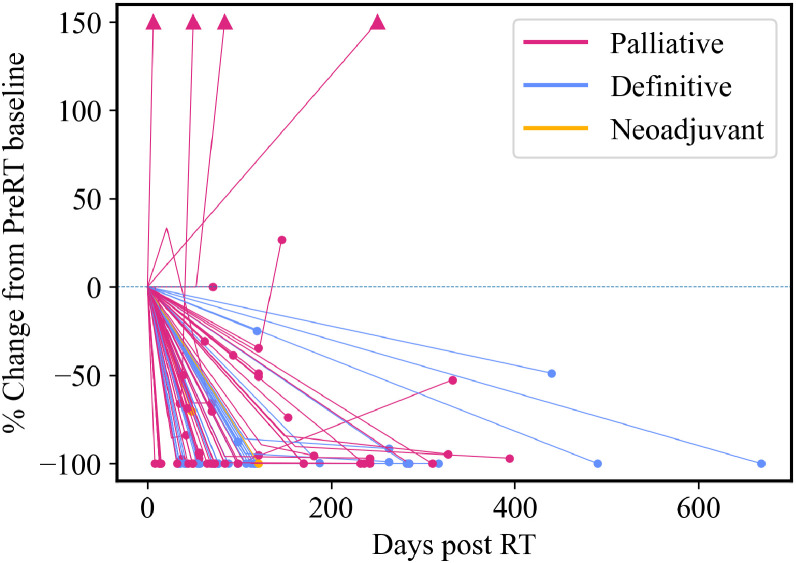
Spider plot showing percent change in local tumor size from baseline (pre-RT) over time among patients with measurable gross disease. Each line represents an individual treated tumor site, and lines are color-coded by treatment intent: palliative (n = 56, magenta), definitive (n = 34, blue), and neoadjuvant (n = 2, orange). Downward trajectories indicate tumor regression, while upward trends show progression. Arrowheads denote sites with >150% growth, truncated for visualization purposes. *Abbreviations:* RT = radiation therapy.

Additionally, we found that the clinical complete response rate for treated gross disease with available size measurements was 62% (n = 57), while an additional 32% (n = 29) of the patients demonstrated a partial but durable response. Notably, local progression, though rare (n = 5; 5%), tended to occur rapidly, and primarily in the palliative setting (Fig. 3). One tumor remained stable over its 71 day observation period (Fig. 3). Among patients treated with adjuvant RT for microscopic disease risk, there was not a statistically significant difference in local disease control based on surgical margin status (*P* = .25; Fig. E4A), acknowledging that positive margins were treated to a slightly higher dose (median 70 Gy BED_10_ vs median 61 Gy BED_10_ for negative margins; *P* = .005). Among patients with more than one treated site (n = 177), 162 (92%) had consistent local control outcomes across all sites, while the remaining 15 (8%) showed mixed responses to RT.

### Distant tumor control

**Figure 4:**
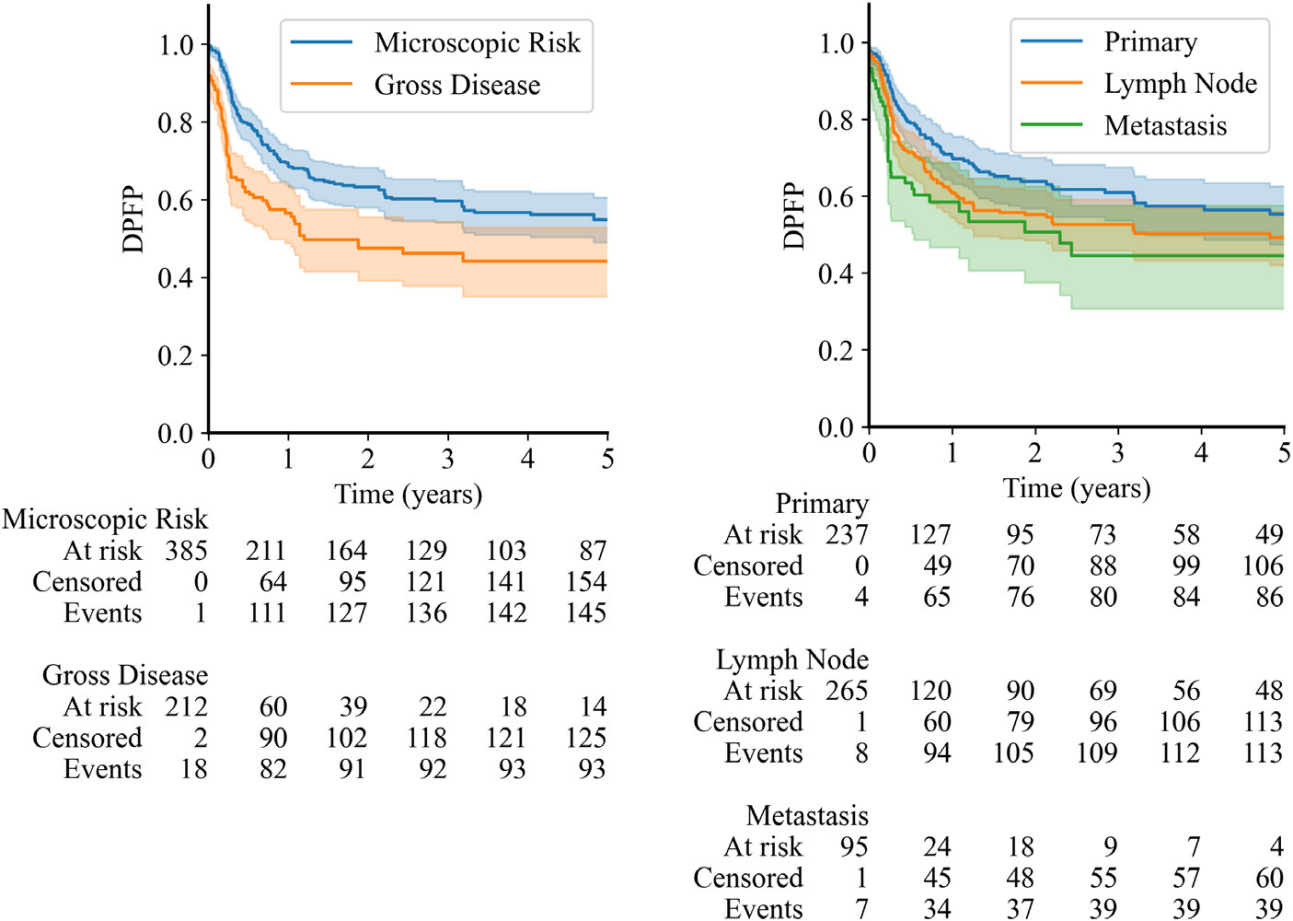
Kaplan-Meier curves showing distant progression-free probability (DPFP). (LEFT) DPFP of sites treated for microscopic disease risk (blue line) and gross disease (orange line) (*P* < .0001). (RIGHT) DPFP for primary tumor sites (blue line), lymph nodes (orange line), and secondary/metastatic (green line) tumor sites treated with radiation therapy. Among pairwise comparisons between groups, the difference between primary and metastatic sites reached statistical significance (Bonferroni-corrected *P* = .014).

We next assessed distant tumor control, understanding that MCC tends to be an aggressive malignancy with high metastatic potential. As anticipated, we observed that distant control rates were lower than local control rates across the entire cohort, with 1- and 3-year DPFP of 65% and 55%, respectively (Fig. E1). Patients treated with RT for gross disease experienced more rapid and frequent distant progression compared to patients treated for microscopic disease risk (1-year DPFP of 56% vs 69%; *P* < .0001) (Fig. 4A), consistent with the generally advanced stage of this subgroup (Table 1, Fig. E5). Patients who received RT at primary tumor site(s) had improved distant control rates compared to those treated for lymph node or other metastatic site(s) with 1- and 3-year DPFP of 71% and 62% versus 62% and 54%,respectively, for lymph nodes, and 59% and 45%, respectively, for metastatic sites (Fig. 4B). Note that we observed no difference in distant control rates for patients with in-transit versus distant metastases (*P* = .79, Fig. E2B). In the microscopic risk group, distant control declined with higher clinical stage, whereas outcomes remained similar across clinical stages in the gross disease setting (Fig. E5). For patients treated with adjuvant RT for microscopic disease, distant control was similar regardless of margin status (*P* = .25, Fig. E4B). To further characterize distant failures, we assessed patterns of recurrence by treated site, with lymph node recurrences more frequent overall compared with other metastases (Fig. E6).

### Overall survival

**Figure 5:**
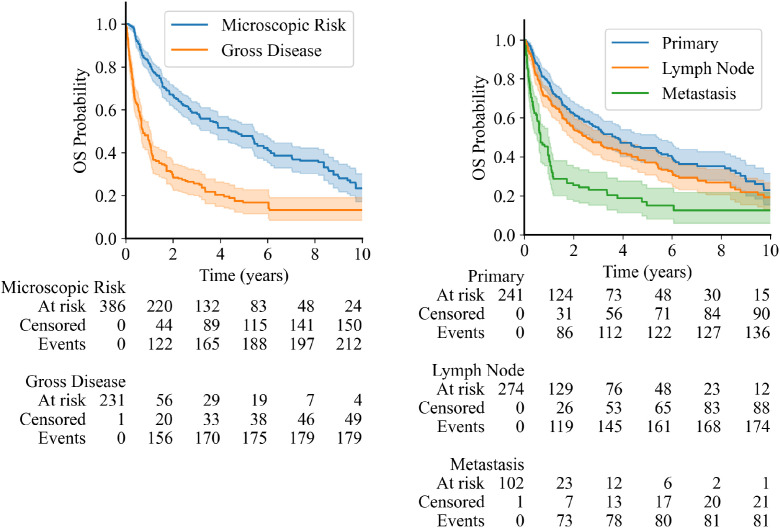
Kaplan-Meier curves showing overall survival (OS) probability. (LEFT) OS for patients with sites treated for microscopic disease risk (blue line) versus gross disease (orange line) (*P* < .0001) and (RIGHT) OS for patients whose treated sites were primary (blue line), lymph nodes (orange line), and secondary/metastatic (green line). Among pairwise comparisons between groups, the differences between primary and metastatic sites and between lymph nodes and metastatic sites were statistically significant (Bonferroni-corrected *P* < .0001).

Given the potential impact of tumor burden on long-term mortality, we next evaluated OS across the cohort. Patients with microscopic disease risk had significantly improved OS compared to those with gross disease (*P* < .0001), with a median OS of 4.77 versus 0.72 years, respectively (Fig. 5A), noting significantly higher clinical stage distribution in the gross disease cohort (Table 1). Patients with localized (stage I-II) or regional (stage III) disease had better OS than patients with distant metastatic (stage IV) disease, particularly in the microscopic disease risk group; however, OS was comparably poor across all clinical stages in patients with gross disease (Fig. E7). Similarly, patients treated for primary tumors had improved median OS compared to those treated for lymph node or other metastatic sites (3.82 vs 2.50 vs 0.66 years, respectively) (Fig. 5B).

### Influence of radiation dose and other clinical features on tumor control

**Figure 6:**
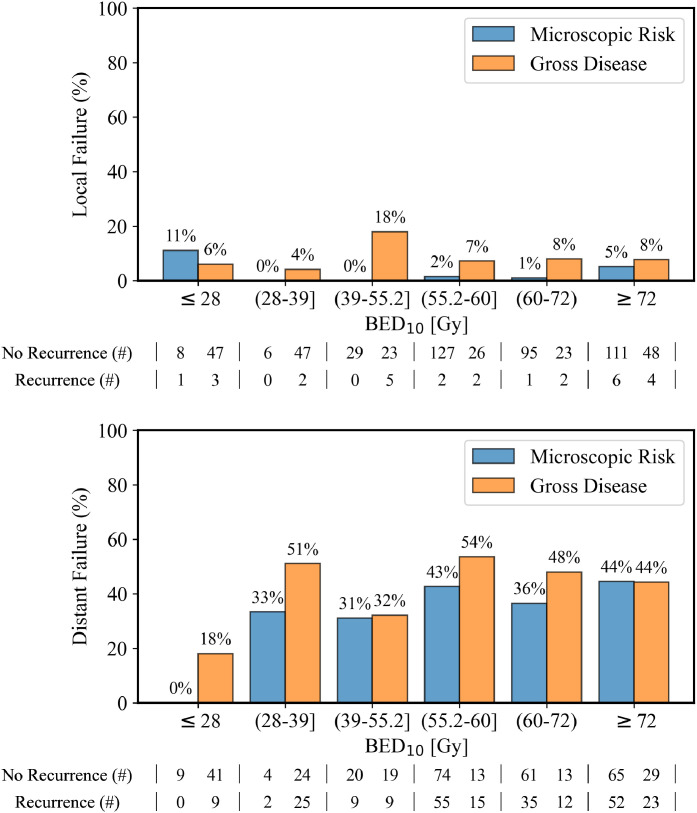
Correlation of radiation dose in BED_10_ with local and distant failure rates. (TOP) Bar plot showing the local failure rate after radiation therapy for tumors treated for microscopic disease risk (blue bars) versus gross tumors (orange bars), categorized by the amount of total radiation delivered in BED_10_. (BOTTOM) Bar plot illustrating the distant failure rate after treatment with radiation therapy for areas of microscopic disease risk (blue bars) and gross disease (orange bars), categorized by the amount of total radiation delivered in BED_10_. The data are stratified by radiation dose ranges, with the x-axis using standard set notation where parentheses “(” indicate exclusivity (value not included) and square brackets “]” indicate inclusivity (value included).

Leveraging the variability in delivered RT dose across our MCC cohort, we next studied how radiation dose—normalized as BED_10_ to account for different fractionation schemes—influences tumor control. We found that local tumor control is excellent across the entire spectrum of delivered radiation doses used, both for sites with gross disease and those treated for microscopic disease risk (Fig. 6A and Figs. E8 and E9), acknowledging the small size of the microscopic disease risk cohort getting lower dose RT (n = 9 for ≤28 Gy BED_10_ and n = 6 for >28 Gy to ≤39 Gy BED_10_). For instance, local tumor control was 90% to 95% in both the highest and lowest RT dose groups (Fig. 6A), acknowledging the notable caveat that the competing risk of death was observed to be higher in the lowest RT dose cohorts with gross disease (Fig. E10). Both univariate and multivariate Cox proportional hazard assessments of radiation dose showed no significant association with LPFP (HR, 0.99; 95% CI, 0.97-1.01; *P* = .22 and HR, 1.00; 95% CI, 0.97-1.02; *P* = .67, respectively; Table 2). Although distant control rates were lower overall, DPFP was also independent of radiation dose (Fig. 6B, Fig. E11, Table 2).

**Table 2:** Univariate and multivariate analysis showing the HR for various factors associated with local and distant progression-free survival

		LPFP				DPFP			
		Univariate analysis		Multivariate analysis		Univariate analysis		Multivariate analysis	
Variable		Adjusted HR (95% CI)	*P* value	Adjusted HR (95% CI)	*P* value	Adjusted HR (95% CI)	*P* value	Adjusted HR (95% CI)	*P* value
Age at diagnosis		1.03 (0.99-1.08)	.09	1.04 (1.00-1.09)	.06	1.00 (0.99-1.02)	.53	1.01 (0.99-1.02)	.37
BED_10_		1.00 (0.97-1.01)	.22	1.00 (0.97-1.02)	.66	1.00 (0.99-1.01)	.91	1.00 (1.00-1.01)	.50
Pre-RT size		1.00 (1.00-1.00)	.04	1.00 (1.00-1.00)	.21	1.00 (1.00-1.00)	.97	1.00 (1.00-1.00)	.44
CCI		0.93 (0.82-1.06)	.29	0.92 (0.79-1.06)	.23	1.00 (0.96-1.05)	.98	1.01 (0.96-1.05)	.80
Immuno-suppression	(Yes: 1/No: 0)	0.43 (0.11-1.60)	.21	0.37 (0.10-1.41)	.14	1.01 (0.69-1.47)	.97	0.88 (0.62-1.25)	.48
Systemic therapy	None	Ref		Ref		Ref		Ref	
	Chemo	0.88 (0.34-2.26)	.79	0.69 (0.25-1.93)	.48	**1.90 (1.40-2.57)**	**<.0001**	**1.57 (1.16-2.12)**	**.004**
	Immuno	1.57 (0.62-4.00)	.35	0.88 (0.32-2.43)	.81	**1.87 (1.28-2.71)**	**.001**	1.30 (0.89-1.91)	.18
	Unknown	0.25 (0.04-1.43)	.12	0.25 (0.04-1.47)	.13	0.69 (0.41-1.15)	.16	0.71 (0.46-1.11)	.13
Cancer stage	I-II	Ref		Ref		Ref		Ref	
	III	1.33 (0.59-3.01)	.50	1.20 (0.49-2.92)	.69	**1.58 (1.15-2.18)**	**.005**	1.17 (0.89-1.54)	.27
	IV	2.36 (0.98-5.71)	.06	1.55 (0.53-4.56)	.43	**2.36 (1.64-3.40)**	**<.0001**	**1.53 (1.08-2.17)**	**.02**
Treated site category	Primary	Ref		Ref		Ref		Ref	
	In-transit	2.24 (0.66-7.63)	.20	1.86 (0.49-7.09)	.36	**1.70 (1.00-2.88)**	**.05**	1.25 (0.72-2.17)	.43
	Lymph node	1.13 (0.54-2.34)	.75	0.92 (0.41-2.08)	.84	1.27 (0.97-1.66)	.08	1.09 (0.85-1.40)	.52
	Metastasis	2.10 (0.71-6.20)	.18	1.23 (0.34-4.52)	.75	**1.64 (1.04-2.58)**	**.03**	1.09 (0.69-1.74)	.70
Tumor site	Head and neck	Ref		Ref		Ref		Ref	
	Upper extremity	1.00 (0.40-2.46)	.99	1.19 (0.45-3.16)	.72	0.74 (0.53-1.05)	.09	0.84 (0.62-1.15)	.28
	Torso	1.44 (0.60-3.46)	.41	0.94 (0.33-2.65)	.91	0.87 (0.61-1.24)	.44	**0.71 (0.51-1.00)**	**.05**
	Lower extremity	0.96 (0.32-2.88)	.94	0.87 (0.28-2.74)	.81	0.76 (0.50-1.16)	.21	0.87 (0.59-1.27)	.47
RT category	Adjuvant RT	Ref		Ref		Ref		Ref	
	Gross disease	**3.40 (1.71-6.76)**	**.0005**	**2.55 (1.06-6.11)**	**.036**	**1.68 (1.30-2.18)**	**<.0001**	1.29 (0.97-1.71)	.08
Margin	Pos:1/Neg:0	3.18 (0.75-13.5)	.12	-	-	0.95 (0.54-1.66)	.84	-	-

Boldface values denote statistical significance (*p* ≤ 0.05). *Abbreviations:* CCI = Charlson comorbidity index; DPFP = distant progression-free probability; HR = hazard ratio; LPFP = local progression-free probability; RT = radiation therapy.

We next examined whether various other clinical features were predictors of local or distant tumor control. The vast majority of clinical factors evaluated were not associated with either local or DPFP in our Cox regression analysis (Table 2). The only factor that reached statistical significance for local progression was the presence of gross disease, which remained significant in both univariate (HR, 3.41; 95% CI, 1.72-6.77; *P* = .0005) and multivariate analyses (HR, 2.64; 95% CI, 1.10-6.36; *P* = .03). However, multiple factors were associated with distant progression; presence of gross disease, use of systemic therapy (both chemotherapy and immunotherapy), in-transit or distant metastasis, and higher clinical stage (III or IV) were all significant in univariate analysis, while use of chemotherapy and highest clinical stage (IV) persisted in multivariate analysis, with treatment site on the torso emerging as associated with reduced risk of distant progression in multivariate analysis alone (Table 2). No other clinical characteristics, including radiation dose, age at diagnosis, Charlson comorbidity index, pretreatment tumor size, or margin status, were statistically significant.

## Discussion

This represents the largest study of disease control outcomes among MCC patients receiving RT to-date, and the first to interrogate a wide spectrum of RT dosimetric impact on MCC-specific outcomes. We did not find evidence in our data to suggest that lower (classically palliative) doses of RT were less effective than higher NCCN-recommended RT doses in achieving local or distant disease control, even in the setting of gross disease. Indeed, although MCC can recur locally despite RT, there is no indication in our cohort that a higher dose prevents these rare recurrences. These findings raise the potential for dose de-escalation in the treatment of MCC. This study also highlights the excellent early local control afforded by RT, even for patients with metastatic disease, and therefore raises the potential to utilize RT more readily in the metastatic setting (ie, for consolidation in addition to palliation).

Although our study confirms prior reports of excellent local tumor control with palliative doses of RT,^17^^,^^18^ it stands in contrast to other studies leveraging OS data from the National Cancer Database (NCDB).^25^ In one NCDB cohort of 2093 MCC patients, RT doses of 40 to <50 Gy, 50 to 55 Gy, and >55 to 70 Gy were equally effective from an OS perspective, whereas a 30 to <40 Gy dose was associated with worse survival.^26^ A second NCDB cohort of 2735 patients found that RT doses of 50 to 57 Gy were associated with improved 3-year OS compared with both lower (30 to <50 Gy) and higher (>57 to 80 Gy) doses.^27^ We note, however, that these NCDB studies excluded lower (<30 Gy) doses, and neglected any assessment of local or distant control rates; moreover, although survival is a meaningful endpoint, which we also report herein, we note that OS is increasingly divorced from MCC-specific outcomes in older populations with medical comorbidities. In another study of local and distant relapse for MCC, stratified by radiation dose, ≥50 Gy was associated with reduced local relapse; however, this cohort may have been underpowered with too few endpoints to draw from, and was characterized by a small dynamic range in dose with no sites treated to <30 Gy.^28^

Our study has several limitations inherent to retrospective analyses using administrative databases. Although the cohort analyzed was national in scope, it is confined to the US veteran population and contains few women, potentially limiting the generalizability of the results to broader, more diverse populations. Additionally, many veterans receive portions of their care outside the VA health care system, potentially resulting in incomplete or missing data in this study. Data missingness and the relatively small number of observed recurrences and lower dose treatments further limit the statistical power and our ability to conduct a robust dose-finding analysis with high confidence. We are also unable to identify causal relationships in these data, noting for instance that the paradoxical association of systemic therapy with increased risk of distant recurrence is likely reflective of selection bias (ie, individuals with advanced disease are more likely to receive systemic therapy) and is likely not a consequence of systemic therapy. Similarly, the low distant recurrence rate associated with the lowest palliative dose among patients with gross disease is likely more reflective of a higher competing risk of death as opposed to tumor recurrence. We must also highlight the heterogeneous nature of our cohort, which introduces interpretive caveats in several cases due to admixtures of clinically disparate scenarios.

Another limitation is the lack of detailed physical dose distribution data (ie, we were unable to review DICOM-RT data or other spatial dose representations), which restricts our ability to definitively determine whether recurrences were in-field (local) or out-of-field (distant). This may introduce rare inaccuracies in our recurrence classification. Furthermore, the use of BED_10_ assumes the validity of the linear-quadratic model and does not account for interindividual variability, such as differences in tumor hypoxia, genetic mutations, immune responses, and other patient-specific nuances that could influence treatment outcomes. Moreover, we did not assess RT-associated toxicities in this cohort and were thus unable to report on clinically meaningful risk/benefit ratios or dose-toxicity relationships (note, however, that clinical experience and previous studies demonstrate increased cutaneous morbidity with increased RT dose). Finally, we did not compare this cohort with outcomes among unirradiated MCC patients. Addressing these limitations in future studies will help refine our understanding of optimal treatment strategies for MCC.

## Conclusions

In this retrospective cohort of US veterans, we find that MCC is a radiosensitive tumor with excellent local response and local control rates, even for gross disease and metastatic tumors. Palliative (low) doses of RT demonstrate acceptable local and distant control outcomes, and we were unable to determine a minimum dose below which MCC-specific outcomes were compromised. Acknowledging several caveats, including the small size of key subgroups (eg, low-dose radiation for microscopic disease) and shorter follow-up duration among patients treated for gross disease, this study supports consideration of dose de-escalation as a reasonable treatment option for MCC. Prospective and confirmatory studies are needed to provide clear evidentiary guidance.

## Disclosures

Reid F. Thompson reports a relationship with the US Department of Veterans Affairs that includes employment and funding grants (VA Clinical Sciences Research & Development Career Development Award 1IK2CX002049-01). The other authors declare that they have no known competing financial interests or personal relationships that could have appeared to influence the work reported in this paper.

## Appendix Supplementary materials

MCC Supplementary Material[72].docx Image, application 1

## Footnotes

Supplementary material associated with this article can be found in the online version at doi:10.1016/j.adro.2026.102029.
