# Supplementary material for: No Association Between Radiation Dose and Clinical Outcomes in Merkel Cell Carcinoma in the Veteran Population
Source: Adv Radiat Oncol. 2026 Mar 17;11(6):102029. doi: 10.1016/j.adro.2026.102029 (PMC13092482; doi:10.1016/j.adro.2026.102029)
Supplement: MCC Supplementary Material[72].docx [file mmc1.docx]

# Supplementary Material

#
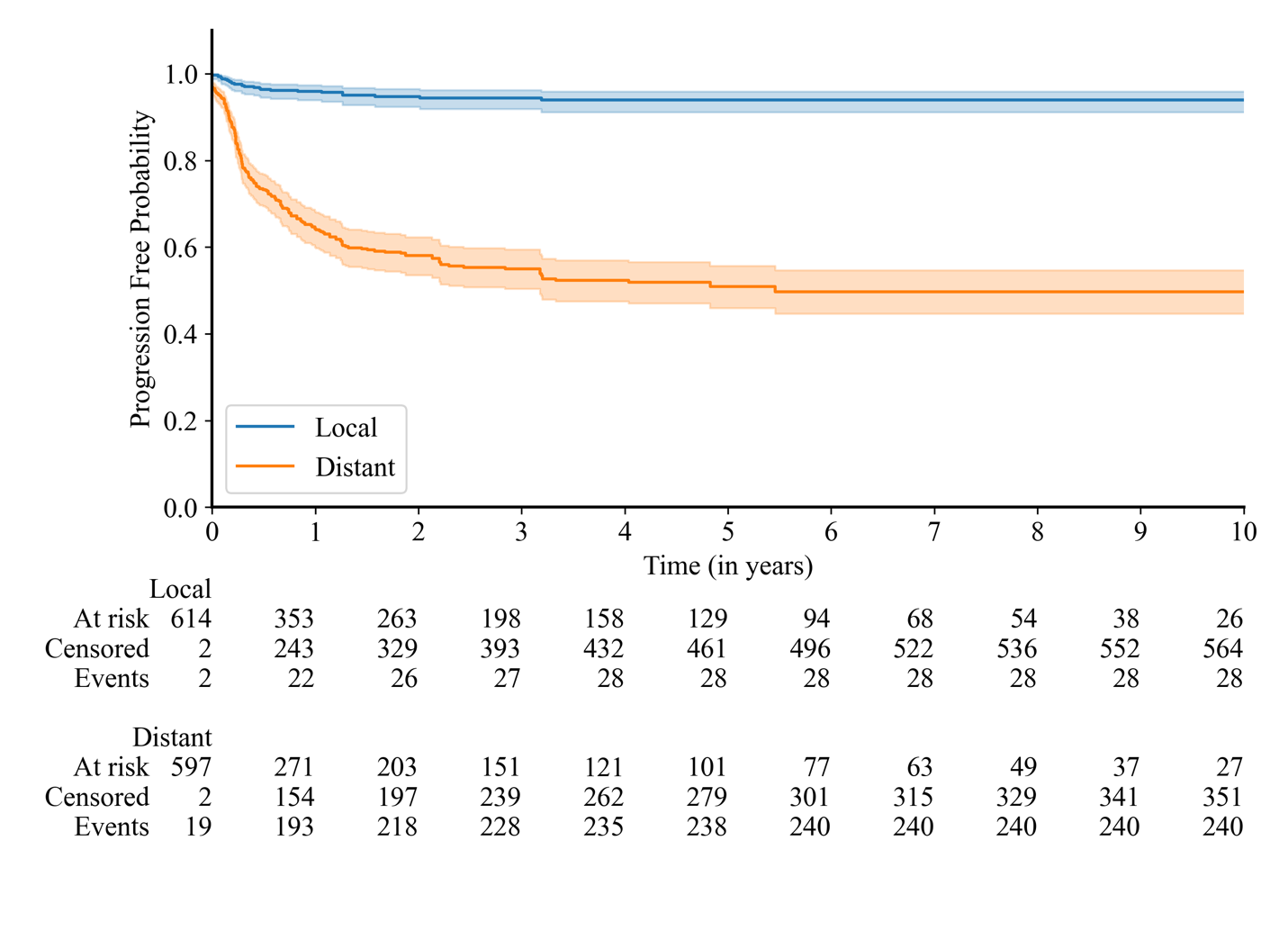


**Figure E1.** Kaplan-Meier curves showing Local Progression Free Probability (LPFP) and Distant Progression free probability (DPFP) of treated tumors for the entire cohort (p=0.0014).

**
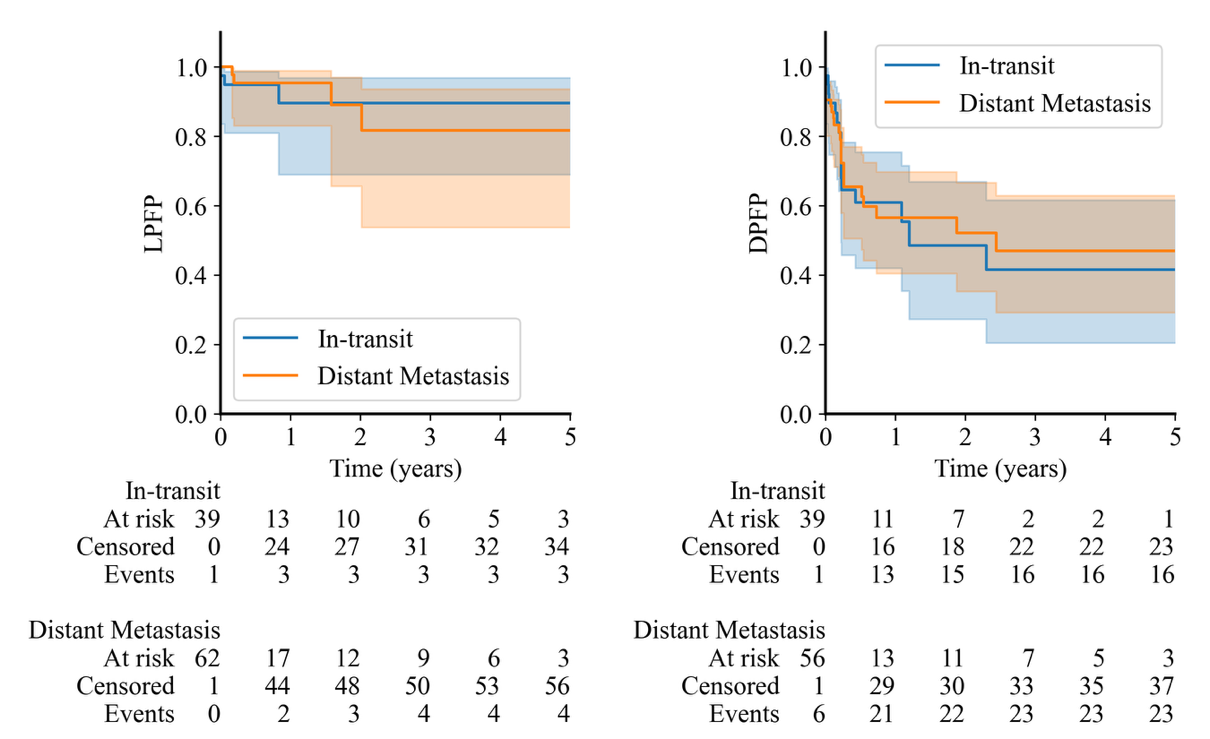
**

**Figure E2.** Kaplan-Meier curves showing (LEFT) Local Progression Free Probability (LPFP, p =0.57) and (RIGHT) Distant Progression Free Probability (DPFP, p=1.00) for tumors treated for metastatic disease grouped as in-transit vs distant metastasis.


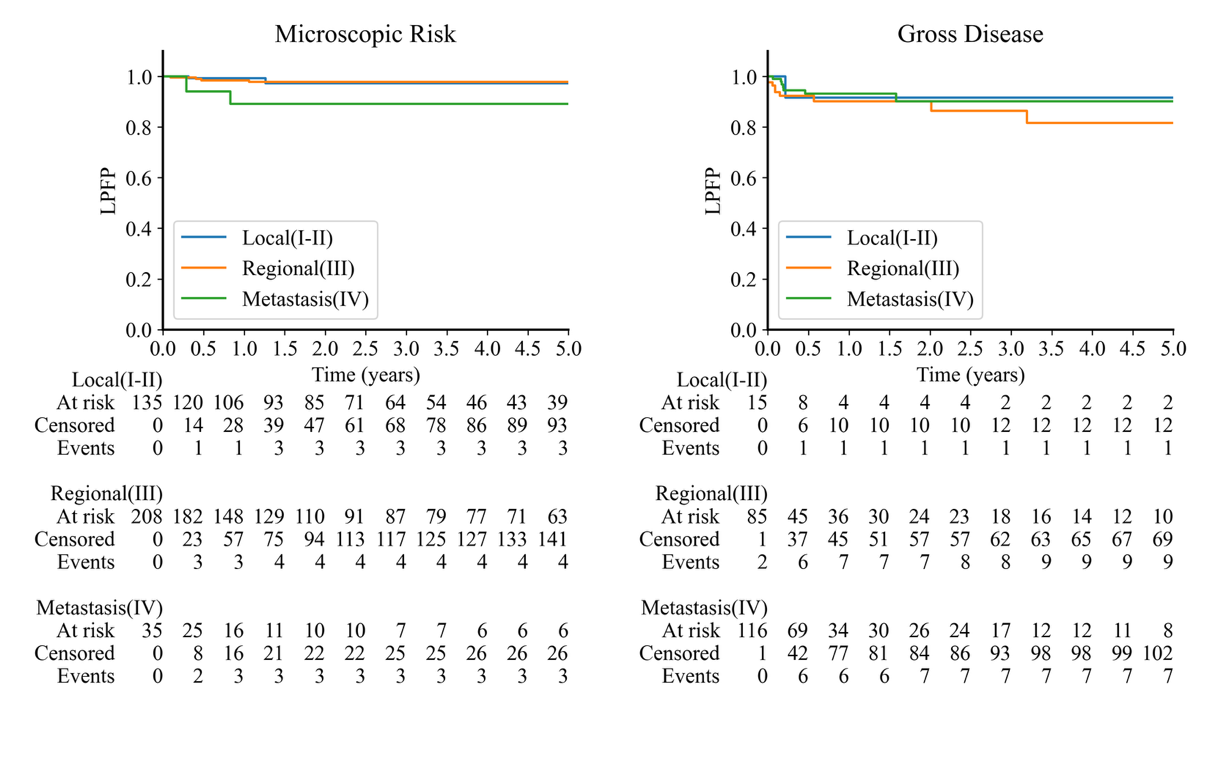


**Figure E3** Local Progression Free Probability (LPFP) for patients with (LEFT) microscopic disease risk after resection and (RIGHT) with gross disease, stratified by their clinical stage at the time of treatment. In both cases, no pairwise comparisons reached statistical significance.


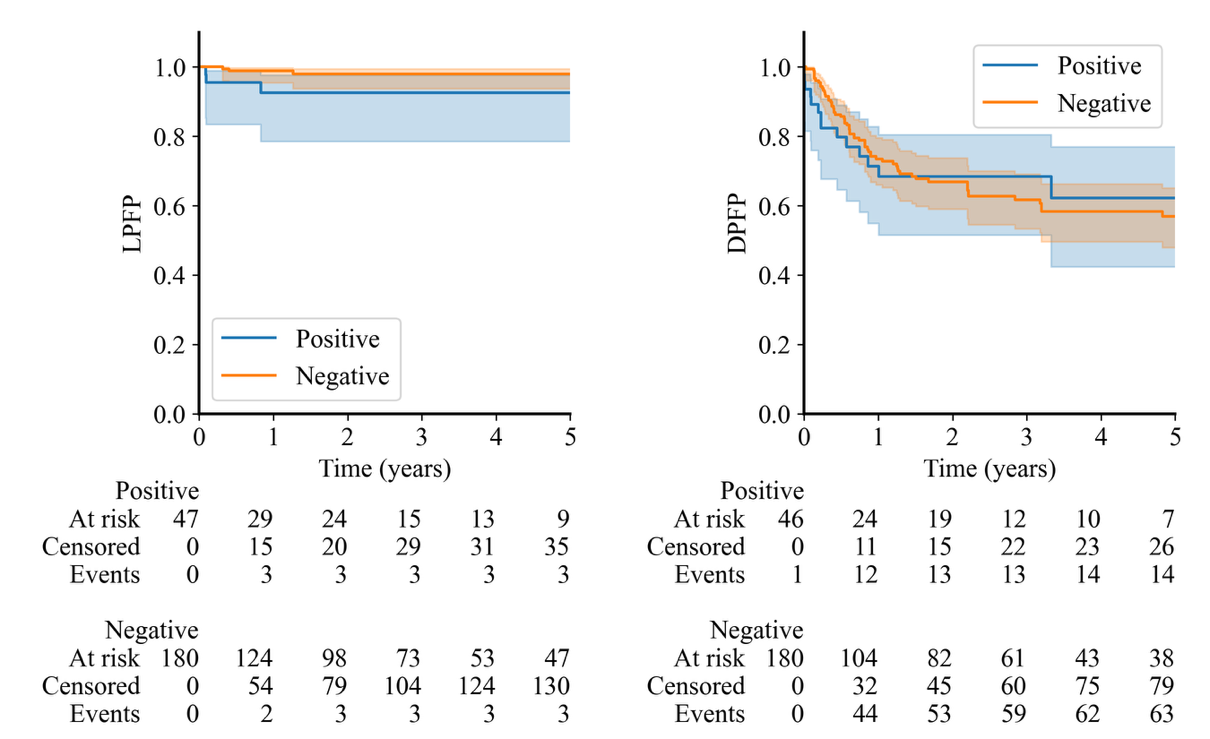


**Figure E4.** Kaplan-Meier curves showing (LEFT) Local Progression Free Probability (LPFP, p =0.25) and (RIGHT) Distant Progression Free Probability (DPFP, p=0.25) for tumors treated for microscopic disease, grouped based on post-surgical margin status.


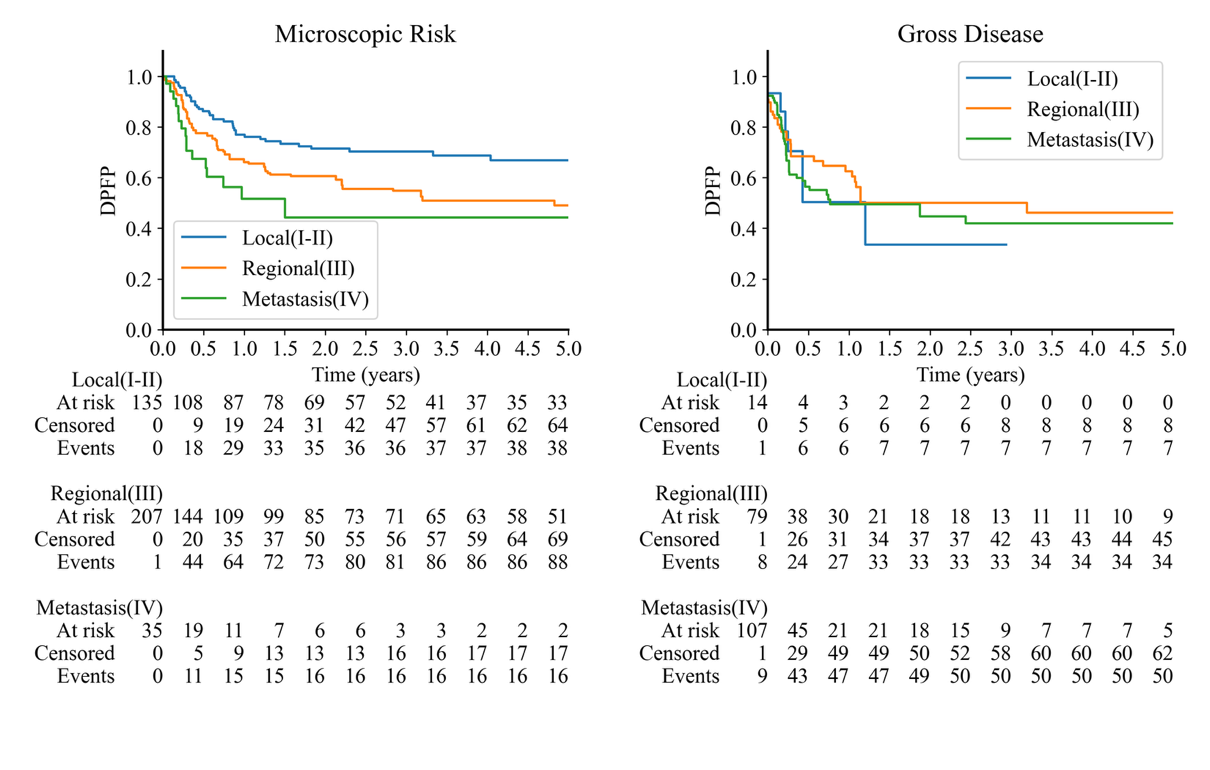


**Figure E5** Distant Progression Free Probability (DPFP) for patients with (LEFT) microscopic disease risk after resection and (RIGHT) with gross disease, stratified by their clinical stage at the time of treatment. Statistically significant differences were observed only within the microscopic disease risk group, between stage I-II vs III and I-II vs IV (Bonferroni-corrected p-values = 0.0096 and 0.005 respectively).


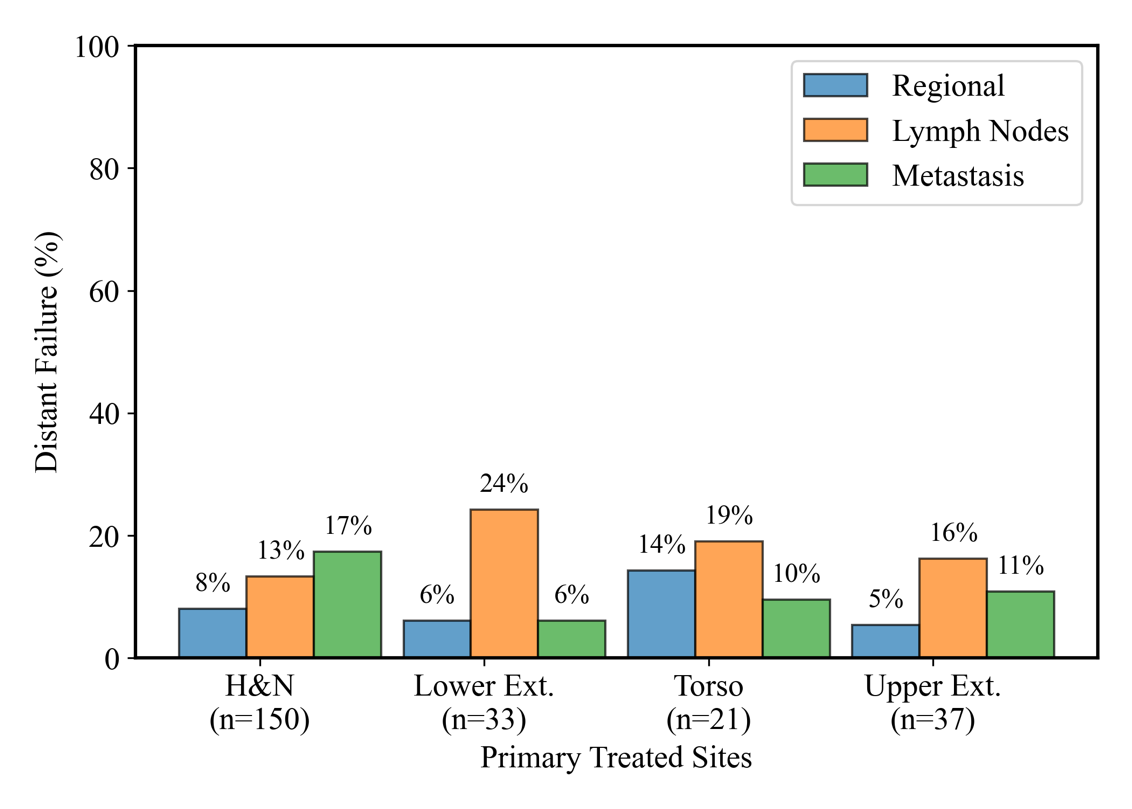


**Figure E6.** Patterns of recurrence across primary treated body regions (head and neck, torso, upper extremity, and lower extremity).


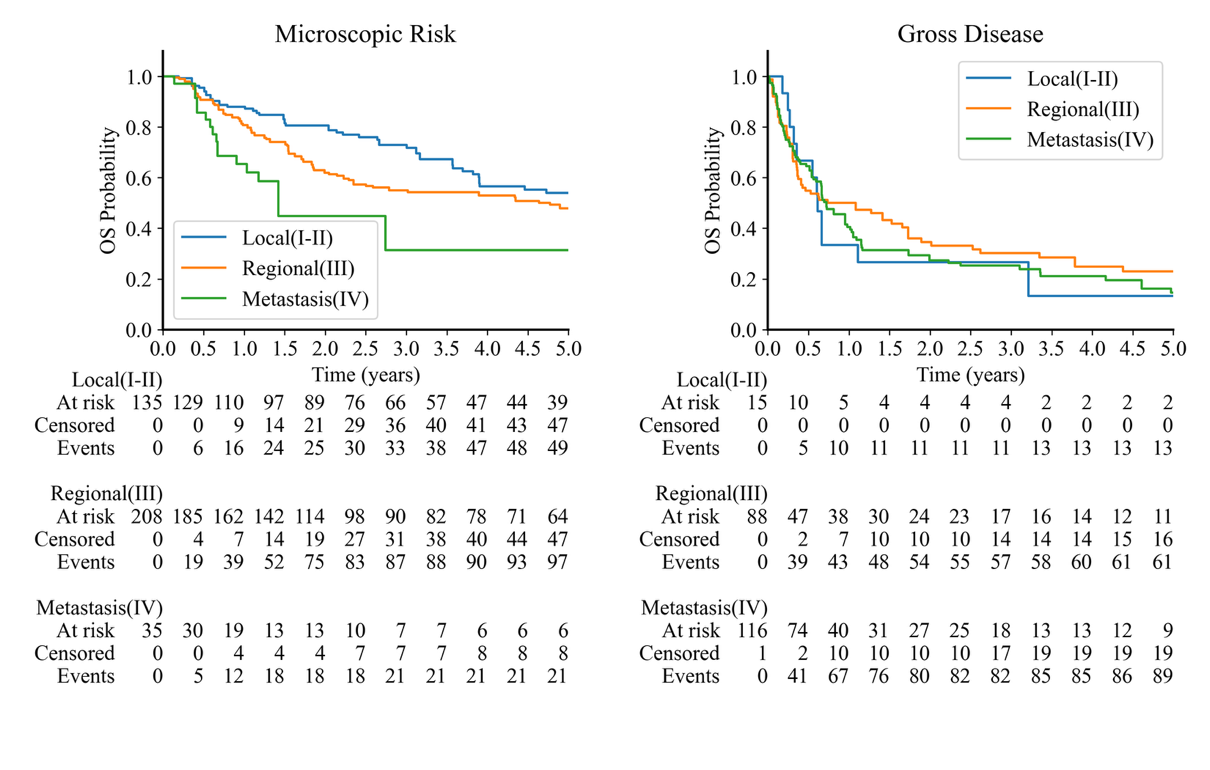


**Figure E7** Overall Survival (OS) for patients with (LEFT) microscopic disease risk after resection and (RIGHT) with gross disease, stratified by their clinical stage at the time of treatment. Statistically significant differences were observed only within the microscopic disease risk group, between stage I-II vs IV and III vs IV (Bonferroni-corrected p-values = 0.0003 and 0.05 respectively).


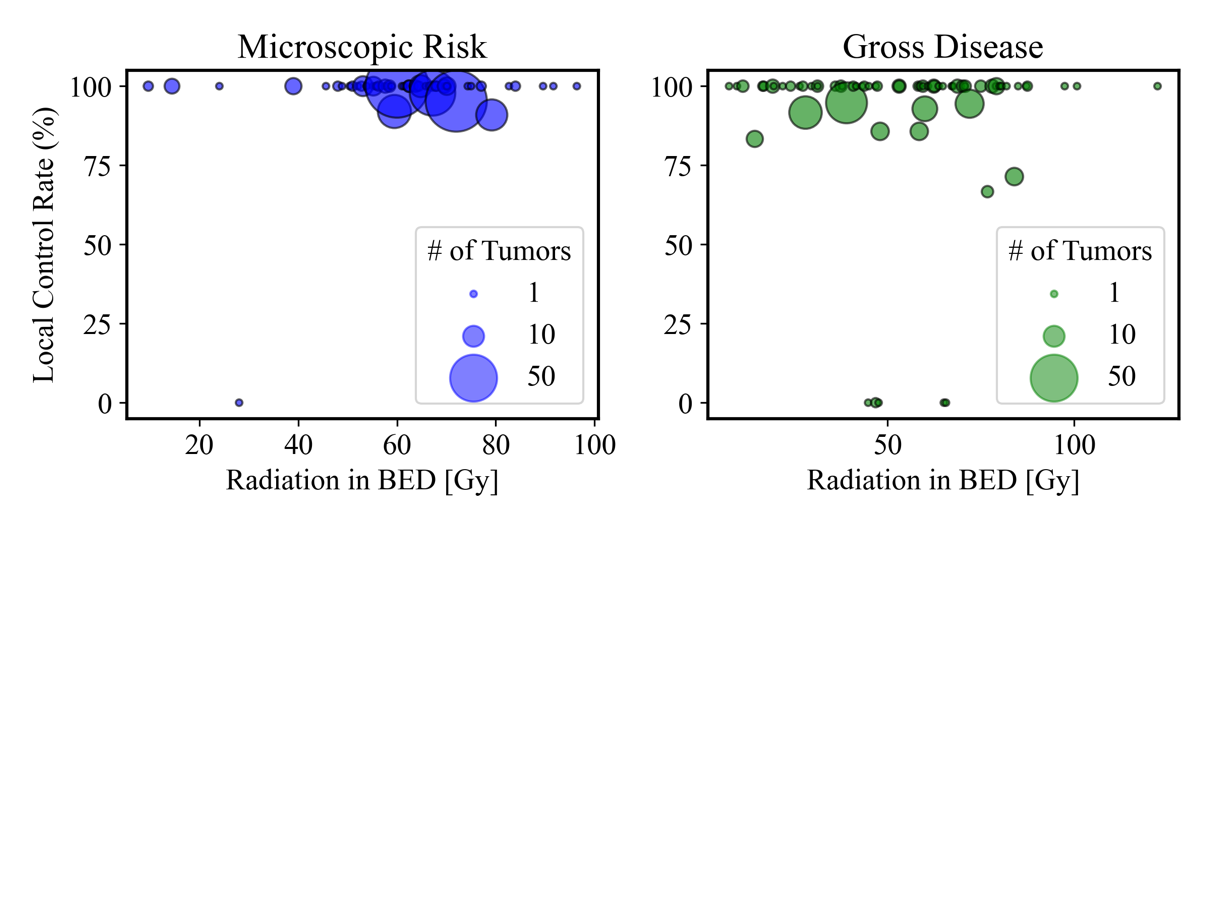


**Figure E8.** Plot showing the local tumor control rate as a function of radiation dose in cases of microscopic disease risk (at left, in blue) and in cases of gross disease (at right, in green).


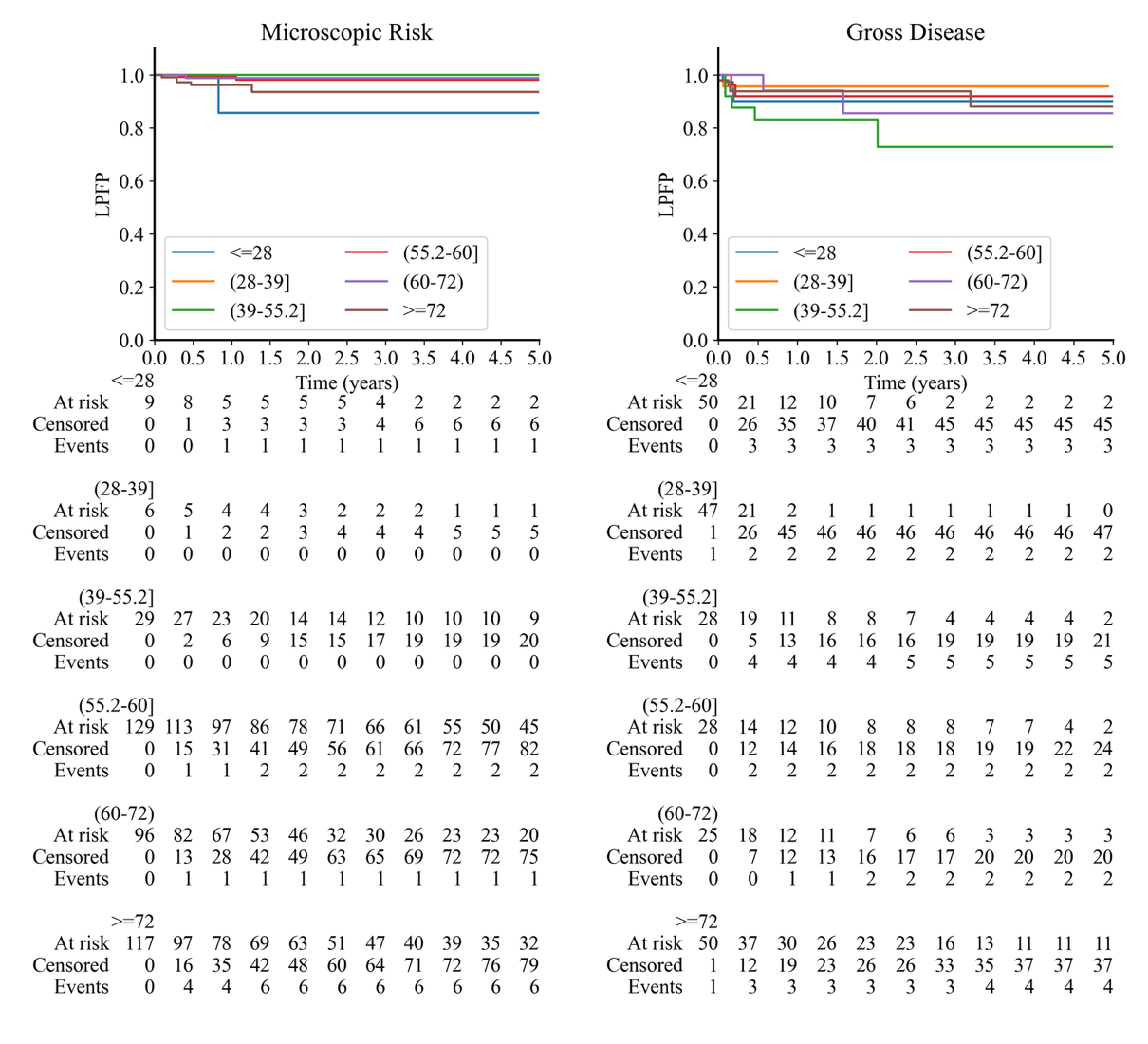


**Figure E9.** Local Progression Free Probability (LPFP) for patients with (LEFT) microscopic disease risk after resection and (RIGHT) with gross disease, stratified by their radiation treatment dose in BED_10_. Parentheses ‘(’ indicate exclusivity (value not included) and square brackets ‘]’ indicate inclusivity (value included).


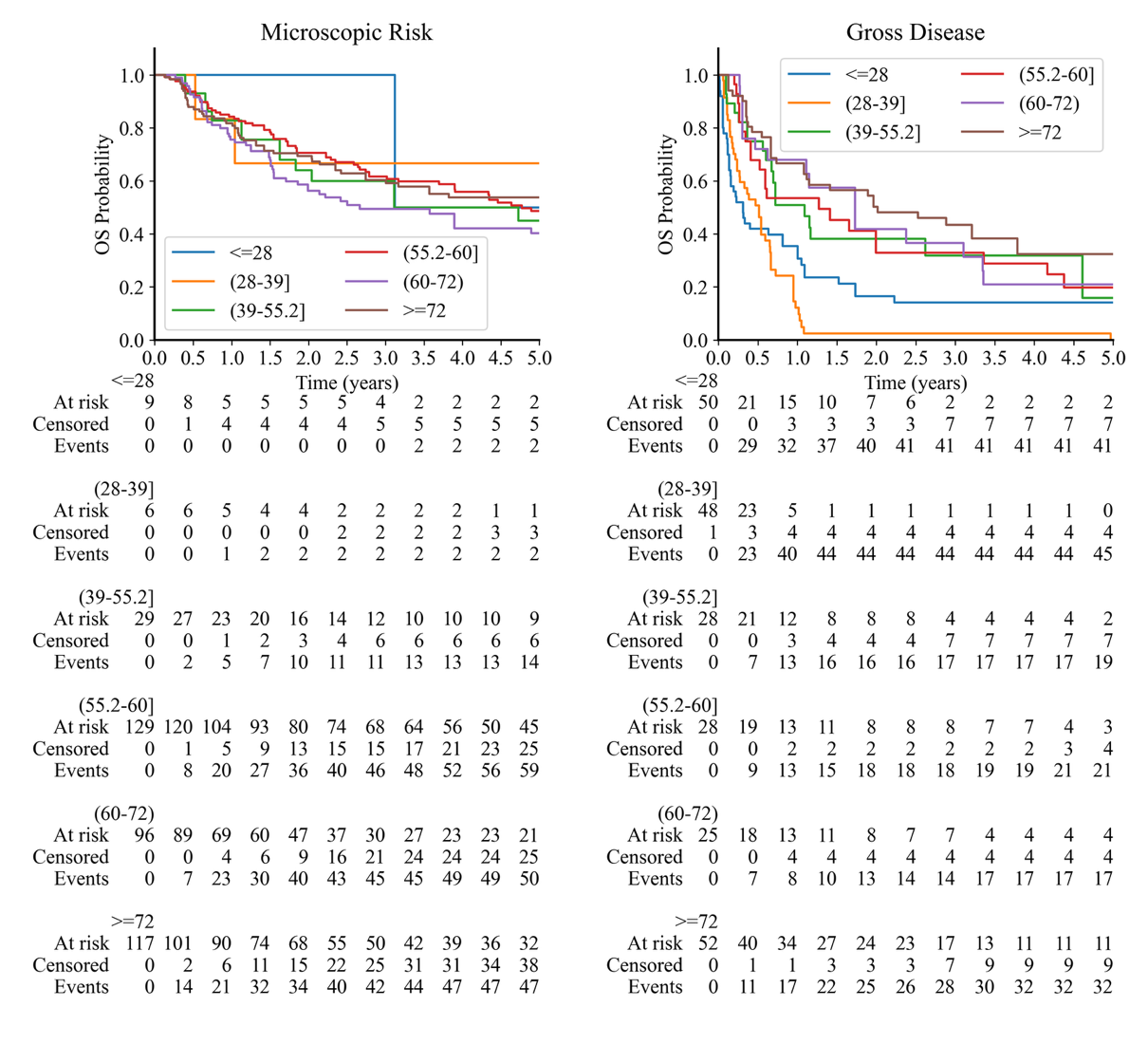


**Figure E10.** Overall Survival (OS) for patients with (LEFT) microscopic disease risk after resection and (RIGHT) with gross disease, stratified by their radiation treatment dose in BED_10_. Parentheses ‘(’ indicate exclusivity (value not included) and square brackets ‘]’ indicate inclusivity (value included).


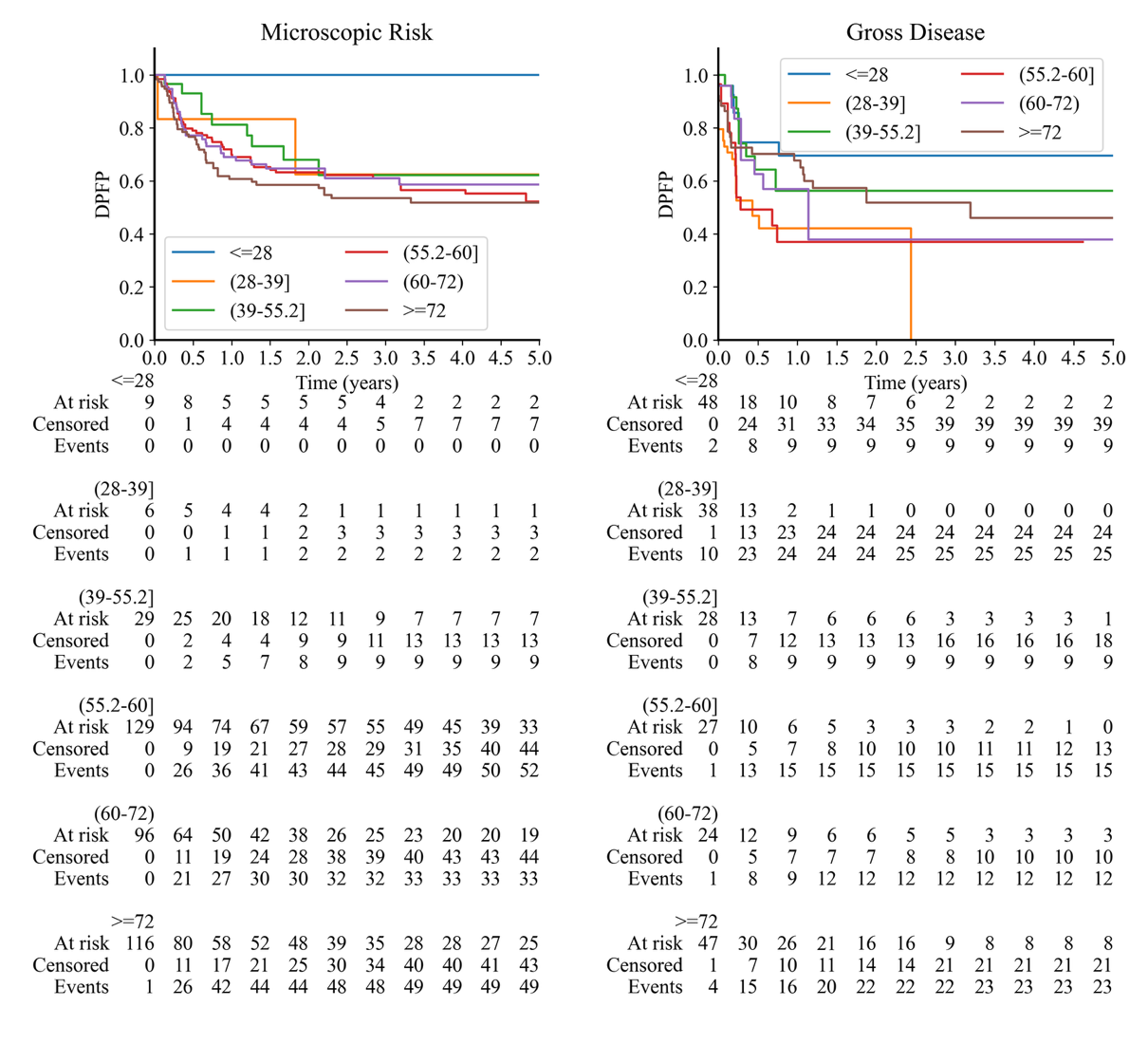


**Figure E11.** Distant Progression Free Probability (DPFP) for patients with (LEFT) microscopic disease risk after resection and (RIGHT) with gross disease, stratified by their radiation treatment dose in BED_10_. Parentheses ‘(’ indicate exclusivity (value not included) and square brackets ‘]’ indicate inclusivity (value included).

**Table E1.** Current Procedural Terminology (CPT) codes used to identify the existence of radiation treatments.

| **CPTCode** | **CPTDescription** |
| --- | --- |
| 19294 | PREPARATION OF TUMOR CAVITY, WITH PLACEMENT OF A RADIATION THERAPY APPLICATOR FOR INTRAOPERATIVE RADIATION THERAPY (IORT) CONCURRENT WITH PARTIAL MASTECTOMY (LIST SEPARATELY IN ADDITION TO CODE FOR PRIMARY PROCEDURE) |
| 19296 | PLACEMENT OF RADIOTHERAPY AFTERLOADING EXPANDABLE CATHETER (SINGLE OR MULTICHANNEL) INTO THE BREAST FOR INTERSTITIAL RADIOELEMENT APPLICATION FOLLOWING PARTIAL MASTECTOMY, INCLUDES IMAGING GUIDANCE; ON DATE SEPARATE FROM PARTIAL MASTECTOMY |
| 19297 | PLACEMENT OF RADIOTHERAPY AFTERLOADING EXPANDABLE CATHETER (SINGLE OR MULTICHANNEL) INTO THE BREAST FOR INTERSTITIAL RADIOELEMENT APPLICATION FOLLOWING PARTIAL MASTECTOMY, INCLUDES IMAGING GUIDANCE; CONCURRENT WITH PARTIAL MASTECTOMY (LIST SEPARATELY IN ADDITION TO CODE FOR PRIMARY PROCEDURE) |
| 19298 | PLACEMENT OF RADIOTHERAPY AFTER LOADING BRACHYTHERAPY CATHETERS (MULTIPLE TUBE AND BUTTON TYPE) INTO THE BREAST FOR INTERSTITIAL RADIOELEMENT APPLICATION FOLLOWING (AT THE TIME OF OR SUBSEQUENT TO) PARTIAL MASTECTOMY, INCLUDES IMAGING GUIDANCE |
| 20555 | PLACEMENT OF NEEDLES OR CATHETERS INTO MUSCLE AND/OR SOFT TISSUE FOR SUBSEQUENT INTERSTITIAL RADIOELEMENT APPLICATION (AT THE TIME OF OR SUBSEQUENT TO THE PROCEDURE) |
| 32553 | PLACEMENT OF INTERSTITIAL DEVICE(S) FOR RADIATION THERAPY GUIDANCE (EG, FIDUCIAL MARKERS, DOSIMETER), PERCUTANEOUS, INTRA-THORACIC, SINGLE OR MULTIPLE |
| 32701 | THORACIC TARGET(S) DELINEATION FOR STEREOTACTIC BODY RADIATION THERAPY (SRS/SBRT), (PHOTON OR PARTICLE BEAM), ENTIRE COURSE OF TREATMENT |
| 41019 | PLACEMENT OF NEEDLES, CATHETERS, OR OTHER DEVICE(S) INTO THE HEAD AND/OR NECK REGION (PERCUTANEOUS, TRANSORAL, OR TRANSNASAL) FOR SUBSEQUENT INTERSTITIAL RADIOELEMENT APPLICATION |
| 49327 | LAPAROSCOPY, SURGICAL; WITH PLACEMENT OF INTERSTITIAL DEVICE(S) FOR RADIATION THERAPY GUIDANCE (EG, FIDUCIAL MARKERS, DOSIMETER), INTRA-ABDOMINAL, INTRAPELVIC, AND/OR RETROPERITONEUM, INCLUDING IMAGING GUIDANCE, IF PERFORMED, SINGLE OR MULTIPLE (LIST SEPARATELY IN ADDITION TO CODE FOR PRIMARY PROCEDURE) |
| 49411 | PLACEMENT OF INTERSTITIAL DEVICE(S) FOR RADIATION THERAPY GUIDANCE (EG, FIDUCIAL MARKERS, DOSIMETER), PERCUTANEOUS, INTRA-ABDOMINAL, INTRA-PELVIC (EXCEPT PROSTATE), AND/OR RETROPERITONEUM, SINGLE OR MULTIPLE |
| 49412 | PLACEMENT OF INTERSTITIAL DEVICE(S) FOR RADIATION THERAPY GUIDANCE (EG, FIDUCIAL MARKERS, DOSIMETER), OPEN, INTRA-ABDOMINAL, INTRAPELVIC, AND/OR RETROPERITONEUM, INCLUDING IMAGE GUIDANCE, IF PERFORMED, SINGLE OR MULTIPLE (LIST SEPARATELY IN ADDITION TO CODE FOR PRIMARY PROCEDURE) |
| 55859 | Transperineal placement of needles or catheters into prostate for interstitial radioelement application, with or without cystoscopy |
| 55860 | Exposure of prostate, any approach, for insertion of radioactive substance |
| 55862 | Exposure of prostate, any approach, for insertion of radioactive substance; with lymph node biopsy(s) (limited pelvic lymphadenectomy) |
| 55865 | Exposure of prostate, any approach, for insertion of radioactive substance; with bilateral pelvic lymphadenectomy, including external iliac, hypogastric and obturator nodes |
| 55875 | Transperineal placement of needles or catheters into prostate for interstitial radioelement application, with or without cystoscopy |
| 55876 | Placement of interstitial device(s) for radiation therapy guidance (eg, fiducial markers, dosimeter), prostate (via needle, any approach), single or multiple |
| 55920 | PLACEMENT OF NEEDLES OR CATHETERS INTO PELVIC ORGANS AND/OR GENITALIA (EXCEPT PROSTATE) FOR SUBSEQUENT INTERSTITIAL RADIOELEMENT APPLICATION |
| 57155 | INSERTION OF UTERINE TANDEM AND/OR VAGINAL OVOIDS FOR CLINICAL BRACHYTHERAPY |
| 57156 | INSERTION OF A VAGINAL RADIATION AFTERLOADING APPARATUS FOR CLINICAL BRACHYTHERAPY |
| 58346 | INSERTION OF HEYMAN CAPSULES FOR CLINICAL BRACHYTHERAPY |
| 58999 | UNLISTED PROCEDURE, FEMALE GENITAL SYSTEM (NONOBSTETRICAL) |
| 61770 | STEREOTACTIC LOCALIZATION, INCLUDING BURR HOLE(S), WITH INSERTION OF CATHETER(S) OR PROBE(S) FOR PLACEMENT OF RADIATION SOURCE |
| 61793 | (When stereotactic radiation therapy is performed jointly by a surgeon and a radiation oncologist [eg, spinal or cranial], the surgeon reports radiosurgery with 61793 |
| 61796 | STEREOTACTIC RADIOSURGERY (PARTICLE BEAM, GAMMA RAY, OR LINEAR ACCELERATOR); 1 SIMPLE CRANIAL LESION |
| 61797 | STEREOTACTIC RADIOSURGERY (PARTICLE BEAM, GAMMA RAY, OR LINEAR ACCELERATOR); EACH ADDITIONAL CRANIAL LESION, SIMPLE (LIST SEPARATELY IN ADDITION TO CODE FOR PRIMARY PROCEDURE) |
| 61798 | STEREOTACTIC RADIOSURGERY (PARTICLE BEAM, GAMMA RAY, OR LINEAR ACCELERATOR); 1 COMPLEX CRANIAL LESION |
| 61799 | STEREOTACTIC RADIOSURGERY (PARTICLE BEAM, GAMMA RAY, OR LINEAR ACCELERATOR); EACH ADDITIONAL CRANIAL LESION, COMPLEX (LIST SEPARATELY IN ADDITION TO CODE FOR PRIMARY PROCEDURE) |
| 61800 | APPLICATION OF STEREOTACTIC HEADFRAME FOR STEREOTACTIC RADIOSURGERY (LIST SEPARATELY IN ADDITION TO CODE FOR PRIMARY PROCEDURE) |
| 63620 | STEREOTACTIC RADIOSURGERY (PARTICLE BEAM, GAMMA RAY, OR LINEAR ACCELERATOR); 1 SPINAL LESION |
| 63621 | STEREOTACTIC RADIOSURGERY (PARTICLE BEAM, GAMMA RAY, OR LINEAR ACCELERATOR); EACH ADDITIONAL SPINAL LESION (LIST SEPARATELY IN ADDITION TO CODE FOR PRIMARY PROCEDURE) |
| 67218 | DESTRUCTION OF LOCALIZED LESION OF RETINA (EG, MACULAR EDEMA, TUMORS), 1 OR MORE SESSIONS; RADIATION BY IMPLANTATION OF SOURCE (INCLUDES REMOVAL OF SOURCE) |
| 73370 | Special medical radiation physics consultation |
| 76370 | Computed tomography guidance for placement of radiation therapy fields |
| 76873 | ULTRASOUND, TRANSRECTAL; PROSTATE VOLUME STUDY FOR BRACHYTHERAPY TREATMENT PLANNING (SEPARATE PROCEDURE) |
| 76950 | ULTRASONIC GUIDANCE FOR PLACEMENT OF RADIATION THERAPY FIELDS |
| 76965 | ULTRASONIC GUIDANCE FOR INTERSTITIAL RADIOELEMENT APPLICATION |
| 77011 | Computed tomography guidance for stereotactic localization |
| 77014 | COMPUTED TOMOGRAPHY GUIDANCE FOR PLACEMENT OF RADIATION THERAPY FIELDS |
| 77261 | THERAPEUTIC RADIOLOGY TREATMENT PLANNING; SIMPLE |
| 77262 | THERAPEUTIC RADIOLOGY TREATMENT PLANNING; INTERMEDIATE |
| 77263 | THERAPEUTIC RADIOLOGY TREATMENT PLANNING; COMPLEX |
| 77280 | THERAPEUTIC RADIOLOGY SIMULATION-AIDED FIELD SETTING; SIMPLE |
| 77285 | THERAPEUTIC RADIOLOGY SIMULATION-AIDED FIELD SETTING; INTERMEDIATE |
| 77290 | THERAPEUTIC RADIOLOGY SIMULATION-AIDED FIELD SETTING; COMPLEX |
| 77293 | RESPIRATORY MOTION MANAGEMENT SIMULATION (LIST SEPARATELY IN ADDITION TO CODE FOR PRIMARY PROCEDURE) |
| 77295 | 3-DIMENSIONAL RADIOTHERAPY PLAN, INCLUDING DOSE-VOLUME HISTOGRAMS |
| 77299 | UNLISTED PROCEDURE, THERAPEUTIC RADIOLOGY CLINICAL TREATMENT PLANNING |
| 77300 | BASIC RADIATION DOSIMETRY CALCULATION, CENTRAL AXIS DEPTH DOSE CALCULATION, TDF, NSD, GAP CALCULATION, OFF AXIS FACTOR, TISSUE INHOMOGENEITY FACTORS, CALCULATION OF NON-IONIZING RADIATION SURFACE AND DEPTH DOSE, AS REQUIRED DURING COURSE OF TREATMENT, ONLY WHEN PRESCRIBED BY THE TREATING PHYSICIAN |
| 77301 | INTENSITY MODULATED RADIOTHERAPY PLAN, INCLUDING DOSE-VOLUME HISTOGRAMS FOR TARGET AND CRITICAL STRUCTURE PARTIAL TOLERANCE SPECIFICATIONS |
| 77305 | TELETHERAPY, ISODOSE PLAN (WHETHER HAND OR COMPUTER CALCULATED); SIMPLE (1 OR 2 PARALLEL OPPOSED UNMODIFIED PORTS DIRECTED TO A SINGLE AREA OF INTEREST) |
| 77306 | TELETHERAPY ISODOSE PLAN; SIMPLE (1 OR 2 UNMODIFIED PORTS DIRECTED TO A SINGLE AREA OF INTEREST), INCLUDES BASIC DOSIMETRY CALCULATION(S) |
| 77307 | TELETHERAPY ISODOSE PLAN; COMPLEX (MULTIPLE TREATMENT AREAS, TANGENTIAL PORTS, THE USE OF WEDGES, BLOCKING, ROTATIONAL BEAM, OR SPECIAL BEAM CONSIDERATIONS), INCLUDES BASIC DOSIMETRY CALCULATION(S) |
| 77310 | TELETHERAPY, ISODOSE PLAN (WHETHER HAND OR COMPUTER CALCULATED); INTERMEDIATE (3 OR MORE TREATMENT PORTS DIRECTED TO A SINGLE AREA OF INTEREST) |
| 77315 | TELETHERAPY, ISODOSE PLAN (WHETHER HAND OR COMPUTER CALCULATED); COMPLEX (MANTLE OR INVERTED Y, TANGENTIAL PORTS, THE USE OF WEDGES, COMPENSATORS, COMPLEX BLOCKING, ROTATIONAL BEAM, OR SPECIAL BEAM CONSIDERATIONS) |
| 77316 | BRACHYTHERAPY ISODOSE PLAN; SIMPLE (CALCULATION[S] MADE FROM 1 TO 4 SOURCES, OR REMOTE AFTERLOADING BRACHYTHERAPY, 1 CHANNEL), INCLUDES BASIC DOSIMETRY CALCULATION(S) |
| 77317 | BRACHYTHERAPY ISODOSE PLAN; INTERMEDIATE (CALCULATION[S] MADE FROM 5 TO 10 SOURCES, OR REMOTE AFTERLOADING BRACHYTHERAPY, 2-12 CHANNELS), INCLUDES BASIC DOSIMETRY CALCULATION(S) |
| 77318 | BRACHYTHERAPY ISODOSE PLAN; COMPLEX (CALCULATION[S] MADE FROM OVER 10 SOURCES, OR REMOTE AFTERLOADING BRACHYTHERAPY, OVER 12 CHANNELS), INCLUDES BASIC DOSIMETRY CALCULATION(S) |
| 77321 | SPECIAL TELETHERAPY PORT PLAN, PARTICLES, HEMIBODY, TOTAL BODY |
| 77326 | BRACHYTHERAPY ISODOSE PLAN; SIMPLE (CALCULATION MADE FROM SINGLE PLANE, 1 TO 4 SOURCES/RIBBON APPLICATION, REMOTE AFTERLOADING BRACHYTHERAPY, 1 TO 8 SOURCES) |
| 77327 | BRACHYTHERAPY ISODOSE PLAN; INTERMEDIATE (MULTIPLANE DOSAGE CALCULATIONS, APPLICATION INVOLVING 5 TO 10 SOURCES/RIBBONS, REMOTE AFTERLOADING BRACHYTHERAPY, 9 TO 12 SOURCES) |
| 77328 | BRACHYTHERAPY ISODOSE PLAN; COMPLEX (MULTIPLANE ISODOSE PLAN, VOLUME IMPLANT CALCULATIONS, OVER 10 SOURCES/RIBBONS USED, SPECIAL SPATIAL RECONSTRUCTION, REMOTE AFTERLOADING BRACHYTHERAPY, OVER 12 SOURCES) |
| 77331 | SPECIAL DOSIMETRY (EG, TLD, MICRODOSIMETRY) (SPECIFY), ONLY WHEN PRESCRIBED BY THE TREATING PHYSICIAN |
| 77332 | TREATMENT DEVICES, DESIGN AND CONSTRUCTION; SIMPLE (SIMPLE BLOCK, SIMPLE BOLUS) |
| 77333 | TREATMENT DEVICES, DESIGN AND CONSTRUCTION; INTERMEDIATE (MULTIPLE BLOCKS, STENTS, BITE BLOCKS, SPECIAL BOLUS) |
| 77334 | TREATMENT DEVICES, DESIGN AND CONSTRUCTION; COMPLEX (IRREGULAR BLOCKS, SPECIAL SHIELDS, COMPENSATORS, WEDGES, MOLDS OR CASTS) |
| 77336 | CONTINUING MEDICAL PHYSICS CONSULTATION, INCLUDING ASSESSMENT OF TREATMENT PARAMETERS, QUALITY ASSURANCE OF DOSE DELIVERY, AND REVIEW OF PATIENT TREATMENT DOCUMENTATION IN SUPPORT OF THE RADIATION ONCOLOGIST, REPORTED PER WEEK OF THERAPY |
| 77338 | MULTI-LEAF COLLIMATOR (MLC) DEVICE(S) FOR INTENSITY MODULATED RADIATION THERAPY (IMRT), DESIGN AND CONSTRUCTION PER IMRT PLAN |
| 77370 | SPECIAL MEDICAL RADIATION PHYSICS CONSULTATION |
| 77371 | RADIATION TREATMENT DELIVERY, STEREOTACTIC RADIOSURGERY (SRS), COMPLETE COURSE |
| 77372 | RADIATION TREATMENT DELIVERY, STEREOTACTIC RADIOSURGERY (SRS), COMPLETE COURSE OF TREATMENT OF CRANIAL LESION(S) CONSISTING OF 1 SESSION; LINEAR ACCELERATOR BASED |
| 77373 | STEREOTACTIC BODY RADIATION THERAPY, TREATMENT DELIVERY, PER FRACTION TO 1 OR MORE LESIONS, INCLUDING IMAGE GUIDANCE, ENTIRE COURSE NOT TO EXCEED 5 FRACTIONS |
| 77380 | PROTON BEAM DELIVERY TO A SINGLE TREATMENT AREA, SINGLE PORT, CUSTOM BLOCK, WITH OR WITHOUT COMPENSATION, WITH TREATMENT SET-UP AND VERIFICATION IMAGES |
| 77381 | PROTON BEAM TREATMENT TO ONE OR TWO TREATMENT AREAS, TWO OR MORE PORTS, TWO OR MORE CUSTOM BLOCKS, AND TWO OR MORE COMPENSATORS, WITH TREATMENT SET-UP AND VERIFICATION IMAGES |
| 77385 | INTENSITY MODULATED RADIATION TREATMENT DELIVERY (IMRT), INCLUDES GUIDANCE AND TRACKING, WHEN PERFORMED; SIMPLE |
| 77386 | INTENSITY MODULATED RADIATION TREATMENT DELIVERY (IMRT), INCLUDES GUIDANCE AND TRACKING, WHEN PERFORMED; COMPLEX |
| 77387 | GUIDANCE FOR LOCALIZATION OF TARGET VOLUME FOR DELIVERY OF RADIATION TREATMENT, INCLUDES INTRAFRACTION TRACKING, WHEN PERFORMED |
| 77399 | UNLISTED PROCEDURE, MEDICAL RADIATION PHYSICS, DOSIMETRY AND TREATMENT DEVICES, AND SPECIAL SERVICES |
| 77400 | DAILY MEGAVOLTAGE TREATMENT MANAGEMENT; SIMPLE |
| 77401 | RADIATION TREATMENT DELIVERY, SUPERFICIAL AND/OR ORTHO VOLTAGE, PER DAY |
| 77402 | RADIATION TREATMENT DELIVERY, >=1 MEV; SIMPLE |
| 77403 | RADIATION TREATMENT DELIVERY, SINGLE TREATMENT AREA, SINGLE PORT OR PARALLEL OPPOSED PORTS, SIMPLE BLOCKS OR NO BLOCKS; 6-10 MEV |
| 77404 | RADIATION TREATMENT DELIVERY, SINGLE TREATMENT AREA, SINGLE PORT OR PARALLEL OPPOSED PORTS, SIMPLE BLOCKS OR NO BLOCKS; 11-19 MEV |
| 77405 | DAILY MEGAVOLTAGE TREATMENT MANAGEMENT; INTERMEDIATE |
| 77406 | RADIATION TREATMENT DELIVERY, SINGLE TREATMENT AREA, SINGLE PORT OR PARALLEL OPPOSED PORTS, SIMPLE BLOCKS OR NO BLOCKS; 20 MEV OR GREATER |
| 77407 | RADIATION TREATMENT DELIVERY, >=1 MEV; INTERMEDIATE |
| 77408 | RADIATION TREATMENT DELIVERY, 2 SEPARATE TREATMENT AREAS, 3 OR MORE PORTS ON A SINGLE TREATMENT AREA, USE OF MULTIPLE BLOCKS; 6-10 MEV |
| 77409 | RADIATION TREATMENT DELIVERY, 2 SEPARATE TREATMENT AREAS, 3 OR MORE PORTS ON A SINGLE TREATMENT AREA, USE OF MULTIPLE BLOCKS; 11-19 MEV |
| 77410 | DAILY MEGAVOLTAGE TREATMENT MANAGEMENT; COMPLEX |
| 77411 | RADIATION TREATMENT DELIVERY, 2 SEPARATE TREATMENT AREAS, 3 OR MORE PORTS ON A SINGLE TREATMENT AREA, USE OF MULTIPLE BLOCKS; 20 MEV OR GREATER |
| 77412 | RADIATION TREATMENT DELIVERY, >=1 MEV; COMPLEX |
| 77413 | RADIATION TREATMENT DELIVERY, 3 OR MORE SEPARATE TREATMENT AREAS, CUSTOM BLOCKING, TANGENTIAL PORTS, WEDGES, ROTATIONAL BEAM, COMPENSATORS, ELECTRON BEAM; 6-10 MEV |
| 77414 | RADIATION TREATMENT DELIVERY, 3 OR MORE SEPARATE TREATMENT AREAS, CUSTOM BLOCKING, TANGENTIAL PORTS, WEDGES, ROTATIONAL BEAM, COMPENSATORS, ELECTRON BEAM; 11-19 MEV |
| 77415 | THERAPEUTIC RADIOLOGY TREATMENT PORT FILM INTERPRETATION AND VERIFICATION, PER TREATMENT COURSE |
| 77416 | RADIATION TREATMENT DELIVERY, 3 OR MORE SEPARATE TREATMENT AREAS, CUSTOM BLOCKING, TANGENTIAL PORTS, WEDGES, ROTATIONAL BEAM, COMPENSATORS, ELECTRON BEAM; 20 MEV OR GREATER |
| 77417 | THERAPEUTIC RADIOLOGY PORT IMAGE(S) |
| 77418 | INTENSITY MODULATED TREATMENT DELIVERY, SINGLE OR MULTIPLE FIELDS/ARCS, VIA NARROW SPATIALLY AND TEMPORALLY MODULATED BEAMS, BINARY, DYNAMIC MLC, PER TREATMENT SESSION |
| 77419 | WEEKLY RADIATION THERAPY MANAGEMENT; CONFORMAL |
| 77420 | WEEKLY RADIATION THERAPY MANAGEMENT; SIMPLE |
| 77421 | STEREOSCOPIC X-RAY GUIDANCE FOR LOCALIZATION OF TARGET VOLUME FOR THE DELIVERY OF RADIATION THERAPY |
| 77422 | HIGH ENERGY NEUTRON RADIATION TREATMENT DELIVERY; SINGLE TREATMENT AREA USING A SINGLE PORT OR PARALLEL-OPPOSED PORTS WITH NO BLOCKS OR SIMPLE BLOCKING |
| 77423 | HIGH ENERGY NEUTRON RADIATION TREATMENT DELIVERY, 1 OR MORE ISOCENTER(S) WITH COPLANAR OR NON-COPLANAR GEOMETRY WITH BLOCKING AND/OR WEDGE, AND/OR COMPENSATOR(S) |
| 77424 | INTRAOPERATIVE RADIATION TREATMENT DELIVERY, X-RAY, SINGLE TREATMENT SESSION |
| 77425 | INTRAOPERATIVE RADIATION TREATMENT DELIVERY, ELECTRONS, SINGLE TREATMENT SESSION |
| 77427 | RADIATION TREATMENT MANAGEMENT, 5 TREATMENTS |
| 77430 | WEEKLY RADIATION THERAPY MANAGEMENT; COMPLEX |
| 77431 | RADIATION THERAPY MANAGEMENT WITH COMPLETE COURSE OF THERAPY CONSISTING OF 1 OR 2 FRACTIONS ONLY |
| 77432 | STEREOTACTIC RADIATION TREATMENT MANAGEMENT OF CRANIAL LESION(S) (COMPLETE COURSE OF TREATMENT CONSISTING OF 1 SESSION) |
| 77435 | STEREOTACTIC BODY RADIATION THERAPY, TREATMENT MANAGEMENT, PER TREATMENT COURSE, TO 1 OR MORE LESIONS, INCLUDING IMAGE GUIDANCE, ENTIRE COURSE NOT TO EXCEED 5 FRACTIONS |
| 77465 | DAILY KILOVOLTAGE TREATMENT MANAGEMENT |
| 77469 | INTRAOPERATIVE RADIATION TREATMENT MANAGEMENT |
| 77470 | SPECIAL TREATMENT PROCEDURE (EG, TOTAL BODY IRRADIATION, HEMIBODY RADIATION, PER ORAL OR ENDOCAVITARY IRRADIATION) |
| 77499 | UNLISTED PROCEDURE, THERAPEUTIC RADIOLOGY TREATMENT MANAGEMENT |
| 77520 | PROTON TREATMENT DELIVERY; SIMPLE, WITHOUT COMPENSATION |
| 77522 | PROTON TREATMENT DELIVERY; SIMPLE, WITH COMPENSATION |
| 77523 | PROTON TREATMENT DELIVERY; INTERMEDIATE |
| 77524 | Proton treatment delivery to one or more treatment areas utilizing two or more ports per treatment area with matching or patching fields and/or multiple isocenters, with custom blocks and compensators |
| 77525 | PROTON TREATMENT DELIVERY; COMPLEX |
| 77600 | HYPERTHERMIA, EXTERNALLY GENERATED; SUPERFICIAL (IE, HEATING TO A DEPTH OF 4 CM OR LESS) |
| 77605 | HYPERTHERMIA, EXTERNALLY GENERATED; DEEP (IE, HEATING TO DEPTHS GREATER THAN 4 CM) |
| 77610 | HYPERTHERMIA GENERATED BY INTERSTITIAL PROBE(S); 5 OR FEWER INTERSTITIAL APPLICATORS |
| 77615 | HYPERTHERMIA GENERATED BY INTERSTITIAL PROBE(S); MORE THAN 5 INTERSTITIAL APPLICATORS |
| 77620 | HYPERTHERMIA GENERATED BY INTRACAVITARY PROBE(S) |
| 77750 | INFUSION OR INSTILLATION OF RADIOELEMENT SOLUTION (INCLUDES 3-MONTH FOLLOW-UP CARE) |
| 77761 | INTRACAVITARY RADIATION SOURCE APPLICATION; SIMPLE |
| 77762 | INTRACAVITARY RADIATION SOURCE APPLICATION; INTERMEDIATE |
| 77763 | INTRACAVITARY RADIATION SOURCE APPLICATION; COMPLEX |
| 77767 | REMOTE AFTERLOADING HIGH DOSE RATE RADIONUCLIDE SKIN SURFACE BRACHYTHERAPY, INCLUDES BASIC DOSIMETRY, WHEN PERFORMED; LESION DIAMETER UP TO 2.0 CM OR 1 CHANNEL |
| 77768 | REMOTE AFTERLOADING HIGH DOSE RATE RADIONUCLIDE SKIN SURFACE BRACHYTHERAPY, INCLUDES BASIC DOSIMETRY, WHEN PERFORMED; LESION DIAMETER OVER 2.0 CM AND 2 OR MORE CHANNELS, OR MULTIPLE LESIONS |
| 77770 | REMOTE AFTERLOADING HIGH DOSE RATE RADIONUCLIDE INTERSTITIAL OR INTRACAVITARY BRACHYTHERAPY, INCLUDES BASIC DOSIMETRY, WHEN PERFORMED; 1 CHANNEL |
| 77771 | REMOTE AFTERLOADING HIGH DOSE RATE RADIONUCLIDE INTERSTITIAL OR INTRACAVITARY BRACHYTHERAPY, INCLUDES BASIC DOSIMETRY, WHEN PERFORMED; 2-12 CHANNELS |
| 77772 | REMOTE AFTERLOADING HIGH DOSE RATE RADIONUCLIDE INTERSTITIAL OR INTRACAVITARY BRACHYTHERAPY, INCLUDES BASIC DOSIMETRY, WHEN PERFORMED; OVER 12 CHANNELS |
| 77776 | INTERSTITIAL RADIATION SOURCE APPLICATION; SIMPLE |
| 77777 | INTERSTITIAL RADIATION SOURCE APPLICATION; INTERMEDIATE |
| 77778 | INTERSTITIAL RADIATION SOURCE APPLICATION, COMPLEX, INCLUDES SUPERVISION, HANDLING, LOADING OF RADIATION SOURCE, WHEN PERFORMED |
| 77781 | REMOTE AFTERLOADING HIGH INTENSITY BRACHYTHERAPY; 1-4 SOURCE POSITIONS OR CATHETERS |
| 77782 | REMOTE AFTERLOADING HIGH INTENSITY BRACHYTHERAPY; 5-8 SOURCE POSITIONS OR CATHETERS |
| 77783 | REMOTE AFTERLOADING HIGH INTENSITY BRACHYTHERAPY; 9-12 SOURCE POSITIONS OR CATHETERS |
| 77784 | REMOTE AFTERLOADING HIGH INTENSITY BRACHYTHERAPY; OVER 12 SOURCE POSITIONS OR CATHETERS |
| 77785 | REMOTE AFTERLOADING HIGH DOSE RATE RADIONUCLIDE BRACHYTHERAPY; 1 CHANNEL |
| 77786 | REMOTE AFTERLOADING HIGH DOSE RATE RADIONUCLIDE BRACHYTHERAPY; 2-12 CHANNELS |
| 77787 | REMOTE AFTERLOADING HIGH DOSE RATE RADIONUCLIDE BRACHYTHERAPY; OVER 12 CHANNELS |
| 77789 | SURFACE APPLICATION OF LOW DOSE RATE RADIONUCLIDE SOURCE |
| 77790 | SUPERVISION, HANDLING, LOADING OF RADIATION SOURCE |
| 77799 | UNLISTED PROCEDURE, CLINICAL BRACHYTHERAPY |
| 79000 | RADIOPHARMACEUTICAL THERAPY, HYPER-THYROIDISM; INITIAL, INCLUDING EVALUATION OF PATIENT |
| 79001 | RADIOPHARMACEUTICAL THERAPY, HYPER-THYROIDISM; SUBSEQUENT, EACH THERAPY |
| 79005 | RADIOPHARMACEUTICAL THERAPY, BY ORAL ADMINISTRATION |
| 79020 | RADIOPHARMACEUTICAL THERAPY, THYROID SUPPRESSION (EUTHYROID CARDIAC DISEASE), INCLUDING EVALUATION OF PATIENT |
| 79030 | RADIOPHARMACEUTICAL ABLATION OF GLAND FOR THYROID CARCINOMA |
| 79035 | RADIOPHARMACEUTICAL THERAPY FOR METASTASES OF THYROID CARCINOMA |
| 79100 | RADIOPHARMACEUTICAL THERAPY, POLYCYTHEMIA VERA, CHRONIC LEUKEMIA, EACH TREATMENT BY INTRAVENOUS INJECTION |
| 79101 | RADIOPHARMACEUTICAL THERAPY, BY INTRAVENOUS ADMINISTRATION |
| 79200 | RADIOPHARMACEUTICAL THERAPY, BY INTRACAVITARY ADMINISTRATION |
| 79300 | RADIOPHARMACEUTICAL THERAPY, BY INTERSTITIAL RADIOACTIVE COLLOID ADMINISTRATION |
| 79400 | RADIOPHARMACEUTICAL THERAPY, NONTHYROID, NONHEMATOLOGIC BY INTRAVENOUS INJECTION |
| 79403 | RADIOPHARMACEUTICAL THERAPY, RADIOLABELED MONOCLONAL ANTIBODY BY INTRAVENOUS INFUSION |
| 79420 | INTRAVASCULAR RADIOPHARMACEUTICAL THERAPY, PARTICULATE |
| 79440 | RADIOPHARMACEUTICAL THERAPY, BY INTRA-ARTICULAR ADMINISTRATION |
| 79445 | RADIOPHARMACEUTICAL THERAPY, BY INTRA-ARTERIAL PARTICULATE ADMINISTRATION |
| 79900 | PROVISION OF THERAPEUTIC RADIOPHARMACEUTICAL(S) |
| 79999 | RADIOPHARMACEUTICAL THERAPY, UNLISTED PROCEDURE |
| 92974 | TRANSCATHETER PLACEMENT OF RADIATION DELIVERY DEVICE FOR SUBSEQUENT CORONARY INTRAVASCULAR BRACHYTHERAPY (LIST SEPARATELY IN ADDITION TO CODE FOR PRIMARY PROCEDURE) |
| 0073T | intensity-modulated radiation therapy (IMRT) treatment delivery |
| 0082T | Stereotactic body radiation therapy, treatment delivery, one or more treatment areas, per day |
| 0083T | Stereotactic body radiation therapy, treatment management, per day |
| 0182T | HIGH DOSE RATE ELECTRONIC BRACHYTHERAPY, PER FRACTION JANUARY 2018 |
| 0190T | PLACEMENT OF INTRAOCULAR RADIATION SOURCE APPLICATOR (LIST SEPARATELY IN ADDITION TO PRIMARY PROCEDURE) |
| 0197T | Intra-fraction localization and tracking of target or patient motion during delivery of radiation therapy (e.g., 3D positional tracking, gating, 3D surface tracking), each fraction of treatment |
| 0394T | HIGH DOSE RATE ELECTRONIC BRACHYTHERAPY, SKIN SURFACE APPLICATION, PER FRACTION, INCLUDES BASIC DOSIMETRY, WHEN PERFORMED |
| 0395T | HIGH DOSE RATE ELECTRONIC BRACHYTHERAPY, INTERSTITIAL OR INTRACAVITARY TREATMENT, PER FRACTION, INCLUDES BASIC DOSIMETRY, WHEN PERFORMED |
| 0520F | RADIATION DOSE LIMITS TO NORMAL TISSUES ESTABLISHED PRIOR TO THE INITIATION OF A COURSE OF 3D CONFORMAL RADIATION FOR A MINIMUM OF 2 TISSUE/ORGAN (ONC) |
| 3318F | PATHOLOGY REPORT CONFIRMING MALIGNANCY DOCUMENTED IN THE MEDICAL RECORD AND REVIEWED PRIOR TO THE INITIATION OF RADIATION THERAPY (ONC) |
| 4163F | PATIENT COUNSELING AT A MINIMUM ON ALL OF THE FOLLOWING TREATMENT OPTIONS FOR CLINICALLY LOCALIZED PROSTATE CANCER: ACTIVE SURVEILLANCE, AND INTERSTITIAL PROSTATE BRACHYTHERAPY, AND EXTERNAL BEAM RADIOTHERAPY, AND RADICAL PROSTATECTOMY, PROVIDED PRIOR TO INITIATION OF TREATMENT (PRCA) |
| 4165F | 3-DIMENSIONAL CONFORMAL RADIOTHERAPY (3D-CRT) OR INTENSITY MODULATED RADIATION THERAPY (IMRT) RECEIVED (PRCA) |
| 4181F | CONFORMAL RADIATION THERAPY RECEIVED (NMA-NO MEASURE ASSOCIATED) |
| 4200F | EXTERNAL BEAM RADIOTHERAPY AS PRIMARY THERAPY TO PROSTATE WITH OR WITHOUT NODAL IRRADIATION (PRCA) |
| 4201F | EXTERNAL BEAM RADIOTHERAPY WITH OR WITHOUT NODAL IRRADIATION AS ADJUVANT OR SALVAGE THERAPY FOR PROSTATE CANCER PATIENT (PRCA) |
| A9513 | LUTETIUM LU 177, DOTATATE, THERAPEUTIC, 1 MILLICURIE |
| A9517 | IODINE I-131 SODIUM IODIDE CAPSULE(S), THERAPEUTIC, PER MILLICURIE |
| A9518 | SUPPLY OF RADIOPHARMACEUTICAL THERAPEUTIC IMAGING AGENT, I-131 SODIUM IODIDE SOLUTION, PER UCI |
| A9523 | SUPPLY OF RADIOPHARMACEUTICAL THERAPEUTIC IMAGING AGENT, YTTRIUM 90 IBRITUMOMAB TIUXETAN, PER MCI |
| A9527 | IODINE I-125, SODIUM IODIDE SOLUTION, THERAPEUTIC, PER MILLICURIE |
| A9530 | IODINE I-131 SODIUM IODIDE SOLUTION, THERAPEUTIC, PER MILLICURIE |
| A9534 | SUPPLY OF RADIOPHARMACEUTICAL THERAPEUTIC IMAGING AGENT, I-131 TOSITUMOMAB, PER MILLICURIE |
| A9543 | YTTRIUM Y-90 IBRITUMOMAB TIUXETAN, THERAPEUTIC, PER TREATMENT DOSE, UP TO 40 MILLICURIES |
| A9545 | IODINE I-131 TOSITUMOMAB, THERAPEUTIC, PER TREATMENT DOSE |
| A9563 | SODIUM PHOSPHATE P-32, THERAPEUTIC, PER MILLICURIE |
| A9564 | CHROMIC PHOSPHATE P-32 SUSPENSION, THERAPEUTIC, PER MILLICURIE |
| A9600 | STRONTIUM SR-89 CHLORIDE, THERAPEUTIC, PER MILLICURIE |
| A9603 | SUPPLY OF THERAPEUTIC RADIOPHARMACEUTICAL, I-131 SODIUM IODIDE CAPSULE, PER MCI |
| A9604 | SAMARIUM SM-153 LEXIDRONAM, THERAPEUTIC, PER TREATMENT DOSE, UP TO 150 MILLICURIES |
| A9605 | SAMARIUM SM-153 LEXIDRONAMM, THERAPEUTIC, PER 50 MILLICURIES |
| A9606 | RADIUM RA-223 DICHLORIDE, THERAPEUTIC, PER MICROCURIE |
| A9699 | RADIOPHARMACEUTICAL, THERAPEUTIC, NOT OTHERWISE CLASSIFIED |
| C1064 | SUPPLY OF RADIOPHARMACEUTICAL THERAPEUTIC IMAGING AGENT, SODIUM IODIDE I-131, CAPSULE, EACH ADDITIONAL MCI |
| C1065 | SUPPLY OF RADIOPHARMACUETICAL THERAPEUTIC IMAGING AGENT, SODIUM IODIDE I-131, SOLUTION, EACH ADDITIONAL MCI |
| C1081 | SUPPLY OF RADIOPHARMACEUTICAL THERAPEUTIC IMAGING AGENT, I-131 TOSITUMOMAB, PER DOSE |
| C1083 | SUPPLY OF RADIOPHARMACEUTICAL THERAPEUTIC IMAGING AGENT, YTTRIUM 90 IBRITUMOMAB TIUXETAN, PER DOSE |
| C1164 | BRACHYTHERAPY SEED, I-125 SEED |
| C1174 | NEEDLE, BRACHYTHERAPY, BARD BRACHYSTAR BRACHYTHERAPY NEEDLE |
| C1188 | SUPPLY OF RADIOPHARMACEUTICAL THERAPEUTIC IMAGING AGENT, SODIUM IODIDE I-131, CAPSULE, PER INITIAL 1-5 MCI |
| C1325 | BRACHYTHERAPY SEED, PALLADIUM-103 SEED |
| C1348 | SUPPLY OF RADIOPHARMACEUTICAL THERAPEUTIC IMAGING AGENT, SODIUM IODIDE I-131, SOLUTION, PER INITIAL 1-6 MCI |
| C1350 | BRACHYTHERAPY, PER SOUCE, PROSTASEED I-125 |
| C1700 | NEEDLE, BRACHYTHERAPY NEEDLE, AUTHENTIC MICK TP BRACHYTHERAPY NEEDLE |
| C1701 | NEEDLE, BRACHYTHERAPY, MEDTEC MT-BT-5201-25 BRACHYTHERAPY NEEDLE |
| C1702 | NEEDLE, BRACHYTHERAPY, WWMT BRACHYTHERAPY NEEDLE |
| C1703 | NEEDLE, BRACHYTHERAPY, MENTOR PROSTATE BRACHYTHERAPY NEEDLE |
| C1704 | NEEDLE, BRACHYTHERAPY, MEDTEC MT-BT-5001-25, MT-BT-5051-25 |
| C1705 | NEEDLE, BRACHYTHERAPY, BEST INDUSTRIES FLEXI NEEDLE BRACHYTHERAPY SEED IMPLANTATION (13G, 14G, 15G, 16G, 17G, 18G), BEST INDUSTRIES PROSTATE BRACHYTHERAPY NEEDLE |
| C1706 | NEEDLE, BRACHYTHERAPY, INDIGO PROSTATE SEEDING NEEDLE |
| C1707 | NEEDLE, BRACHYTHERAPY, VARISOURCE INTERSTITIAL IMPLANT NEEDLE |
| C1708 | NEEDLE, BRACHYTHERAPY, UROMED PROSTATE SEEDING NEEDLE |
| C1709 | NEEDLE, BRACHYTHERAPY, REMINGTON MEDICAL BRACHYTHERAPY NEEDLE |
| C1710 | NEEDLE, BRACHYTHERAPY, US BIOPSY PROSTATE SEEDING NEEDLE |
| C1711 | NEEDLE, BRACHYTHERAPY, MD TECH P.S.S. PROSTATE SEEDING SET (NEEDLE) |
| C1712 | NEEDLE, BRACHYTHERAPY, IMAGYN MEDICAL TECHNOLOGIES ISOSTAR PROSTATE BRACHYTHERAPY NEEDLE |
| C1715 | BRACHYTHERAPY NEEDLE |
| C1716 | BRACHYTHERAPY SOURCE, NON-STRANDED, GOLD-198, PER SOURCE |
| C1717 | BRACHYTHERAPY SOURCE, NON-STRANDED, HIGH DOSE RATE IRIDIUM-192, PER SOURCE |
| C1718 | BRACHYTHERAPY SOURCE, IODINE 125, PER SOURCE |
| C1719 | BRACHYTHERAPY SOURCE, NON-STRANDED, LOW DOSE RATE IRIDIUM 192, PER SOURCE |
| C1720 | BRACHYTHERAPY SOURCE, PALLADIUM 103, PER SOURCE |
| C1728 | CATHETER, BRACHYTHERAPY SEED ADMINISTRATION |
| C1790 | BRACHYTHERAPY SEED, NUCLETRON IRIDIUM 192 HDR |
| C1791 | BRACYTHERAPY SEED, NYCOMED AMERSHAM I-125 (ONCOSEED, RAPID STRAND) |
| C1792 | BRACHYTHERAPY SEED, UROMED SYMMETRA I-125 |
| C1793 | BRACHYTHERAPY SEED, BARD INTERSOURCE 103 PALLADIUM SEED 1031L, 1031C |
| C1794 | BRACHYTHERAPY SEED, BARD ISOSEED 103 PALLADIUM SEED PD3S111L, PD3S111P |
| C1795 | BRACHYTHERAPY SEED, BARD BRACHYSOURCE 125 IODINE SEED 1251L, 1251C |
| C1796 | BRACHYTHERAPY SEED, SOURCE TECH MEDICAL I-125 SEED MODEL STM 1251 |
| C1797 | BRACHYTHERAPY SEED, DRAXIMAGE I-125 SEED MODEL LS-1 |
| C1798 | BRACHYTHERAPY SEED, SYNCOR I-125 PHARMASEED MODEL BT-125-1 |
| C1799 | BRACHYTHERAPY SEED, I-PLANT IODINE 125 MODEL 3500 |
| C1800 | BRACHYTHERAPY SEED, MENTOR PDGOLD PD-103 |
| C1801 | BRACHYTHERAPY SEED, MENTOR IOGOLD I-125 |
| C1802 | BRACHYTHERAPY SEED, BEST INDUSTRIES IRIDIUM 192 |
| C1803 | BRACHYTHERAPY SEED, BEST INDUSTRIES IODINE 125 |
| C1804 | BRACHYTHERAPY SEED, BEST INDUSTRIES PALLADIUM 103 |
| C1805 | BRACHYTHERAPY SEED, IMAGYN ISOSTAR IODINE-125 INTERSTITIAL BRACHYTHERAPY SEED |
| C1806 | BRACHYTHERAPY SEED, BEST INDUSTRIES GOLD 198 |
| C2616 | BRACHYTHERAPY SOURCE, NON-STRANDED, HIGH DOSE RATE Y-90, PER SOURCE |
| C2632 | BRACHYTHERAPY SOLUTION, IODINE-125, PER MCI |
| C2633 | BRACHYTHERAPY SOURCE, CESIUM-131, PER SOURCE |
| C2634 | BRACHYTHERAPY SOURCE, NON-STRANDED, HIGH DOSE RATE I-125>1.01MCI, PER SOURCE |
| C2635 | BRACHYTHERAPY SOURCE, NON-STRANDED, HIGH DOSE RATE PD-103>2.2MCI, PER SOURCE |
| C2636 | BRACHYTHERAPY SOURCE, NON-STRANDED, HIGH DOSE RATE PD-103, PER 1MM |
| C2637 | BRACHYTHERAPY SOURCE, NON-STRANDED, HIGH DOSE RATE YB-169, PER SOURCE |
| C2638 | BRACHYTHERAPY SOURCE, STRANDED, LOW DOSE RATE I-125, PER SOURCE |
| C2639 | BRACHYTHERAPY SOURCE, NON-STRANDED, I-125, PER SOURCE |
| C2640 | BRACHYTHERAPY SOURCE, STRANDED, PD-103, PER SOURCE |
| C2641 | BRACHYTHERAPY SOURCE, NON-STRANDED, PD-103, PER SOURCE |
| C2642 | BRACHYTHERAPY SOURCE, STRANDED, CS-131, PER SOURCE |
| C2643 | BRACHYTHERAPY SOURCE, NON-STRANDED, CS-131, PER SOURCE |
| C2644 | BRACHYTHERAPY SOURCE, CESIUM-131 CHLORIDE SOLUTION, PER MILLICURIE |
| C2645 | BRACHYTHERAPY PLANAR SOURCE, PALLADIUM-103, PER SQUARE MILLIMETER |
| C2698 | BRACHYTHERAPY SOURCE, STRANDED, NOS, PER SOURCE |
| C2699 | BRACHYTHERAPY SOURCE, NON-STRANDED, NOS, PER SOURCE |
| C9401 | SUPPLY OF THERAPEUTIC RADIOPHARMACEUTICAL, STRONTIUM-89 CHLORIDE, BRAND NAME, PER MCI |
| C9402 | SUPPLY OF RADIOPHARMACEUTICAL THERAPEUTIC IMAGING AGENT, I-131 SODIUM IODIDE CAPSULE, BRAND NAME, PER MCI |
| C9405 | SUPPLY OF RADIOPHARMACEUTICAL THERAPEUTIC AGENT, I-131 SODIUM IODIDE SOLUTION, BRAND NAME, PER MILLICURIE |
| C9408 | IODINE I-131 IOBENGUANE, THERAPEUTIC, 1 MILLICURIE |
| C9702 | CHECKMATE INTRAVASCULAR BRACHYTHERAPY SYSTEM |
| C9714 | PLACEMENT OF BALLOON CATHETER INTO THE BREAST FOR INTERSTITIAL RADIATION THERAPY FOLLOWING A PARTIAL MASTECTOMY; CONCURRENT/IMMEDIATE (ADD-ON) |
| C9715 | PLACEMENT OF BALLOON CATHETER INTO THE BREAST FOR INTERSTITIAL RADIATION THERAPY FOLLOWING A PARTIAL MASTECTOMY; DELAYED |
| C9725 | PLACEMENT OF APPLICATOR INTO RECTUM FOR HIGH INTENSITY BRACHYTHERAPY |
| C9726 | PLACEMENT AND REMOVAL (IF PERFORMED) OF APPLICATOR INTO BREAST FOR INTRAOPERATIVE RADIATION THERAPY, ADD-ON TO PRIMARY BREAST PROCEDURE |
| C9728 | PLACEMENT OF INTERSTITIAL DEVICE(S) FOR RADIATION THERAPY/SURGERY GUIDANCE (E.G., FIDUCIAL MARKERS, DOSIMETER), FOR OTHER THAN THE FOLLOWING SITES (ANY APPROACH): ABDOMEN, PELVIS, PROSTATE, RETROPERITONEUM, THORAX, SINGLE OR MULTIPLE |
| G0173 | Linear accelerator based stereotactic radiosurgery, complete course of therapy in one session |
| G0174 | IMRT - INTENSITY MODULATED RADIATION THERAPY PLAN, PER SESSION |
| G0178 | INTENSITY MODULATED RADIATION THERAPY (IMRT) DELIVERY TO MULTIPLE AREAS WITH TREATMENT SETUP AND VERIFICATION IMAGES |
| G0242 | MULTI-SOURCE PHOTON STEREOTACTIC RADIOSURGERY (COBALT 60 MULTI-SOURCE CONVERGING BEAMS) PLAN, INCLUDING DOSE VOLUME HISTOGRAMS FOR TARGET AND CRITICAL STRUCTURE TOLERANCES, PLAN OPTIMIZATION PERFORMED FOR HIGHLY CONFORMAL DISTRIBUTIONS, PLAN POSITIONAL AC |
| G0243 | MULTI-SOURCE PHOTON STEREOTACTIC RADIOSURGERY, DELIVERY INCLUDING COLLIMATOR CHANGES AND CUSTOM PLUGGING, COMPLETE COURSE OF TREATMENT, ALL LESIONS |
| G0251 | Linear accelerator based stereotactic radiosurgery, delivery including collimator changes and custom plugging, fractionated treatment, all lesions, per session, maximum five sessions per course of treatment |
| G0256 | Prostate brachytherapy using permanently implanted palladium seeds, including transperitoneal placement of needles or catheters into the prostate, cystoscopy and application of permanent interstitial radiation source |
| G0261 | Prostate brachytherapy using permanently implanted iodine seeds, including transperineal placement of needles or catheters into the prostate, cystoscopy and application of permanent interstitial radiation source |
| G0273 | RADIOPHARMACEUTICAL BIODISTRIBUTION, SINGLE OR MULTIPLE SCANS ON ONE OR MORE DAYS, PRE-TREATMENT PLANNING FOR RADIOPHARMACEUTICAL THERAPY OF NON-HODGKIN'S LYMPHOMA, INCLUDES ADMINISTRATION OF RADIOPHARMACEUTICAL (E.G., RADIOLABELED ANTIBODIES) |
| G0274 | RADIOPHARMACEUTICAL THERAPY, NON-HODGKIN'S LYMPHOMA, INCLUDES ADMINISTRATION OF RADIOPHARMACEUTICAL (.E.G. RADIOLABELED ANTIBODIES) |
| G0338 | Performed for highly conformal distributions, plan positional accuracy and dose linear-accelerator-based stereotactic radiosurgery plan, including dose volume histograms for target and critical structure tolerances, plan optimization verification, all lesions treated, per course of treatment |
| G0339 | Image-guided robotic linear accelerator-based stereotactic radiosurgery, complete course of therapy in one session or first session of fractionated treatment |
| G0340 | Image-guided robotic linear accelerator-based stereotactic radiosurgery, delivery including collimator changes and custom plugging, fractionated treatment, all lesions, per session, second through fifth sessions, maximum five sessions per course of treatm |
| G0458 | Low dose rate (ldr) prostate brachytherapy services, composite rate |
| G6001 | ULTRASONIC GUIDANCE FOR PLACEMENT OF RADIATION THERAPY FIELDS |
| G6002 | STEREOSCOPIC X-RAY GUIDANCE FOR LOCALIZATION OF TARGET VOLUME FOR THE DELIVERY OF RADIATION THERAPY |
| G6003 | RADIATION TREATMENT DELIVERY, SINGLE TREATMENT AREA,SINGLE PORT OR PARALLEL OPPOSED PORTS, SIMPLE BLOCKS OR NO BLOCKS: UP TO 5 MEV |
| G6004 | RADIATION TREATMENT DELIVERY, SINGLE TREATMENT AREA,SINGLE PORT OR PARALLEL OPPOSED PORTS, SIMPLE BLOCKS OR NO BLOCKS: 6-10 MEV |
| G6005 | RADIATION TREATMENT DELIVERY, SINGLE TREATMENT AREA,SINGLE PORT OR PARALLEL OPPOSED PORTS, SIMPLE BLOCKS OR NO BLOCKS: 11-19 MEV |
| G6006 | RADIATION TREATMENT DELIVERY, SINGLE TREATMENT AREA,SINGLE PORT OR PARALLEL OPPOSED PORTS, SIMPLE BLOCKS OR NO BLOCKS: 20 MEV OR GREATER |
| G6007 | RADIATION TREATMENT DELIVERY, 2 SEPARATE TREATMENT AREAS, 3 OR MORE PORTS ON A SINGLE TREATMENT AREA, USE OF MULTIPLE BLOCKS: UP TO 5 MEV |
| G6008 | RADIATION TREATMENT DELIVERY, 2 SEPARATE TREATMENT AREAS, 3 OR MORE PORTS ON A SINGLE TREATMENT AREA, USE OF MULTIPLE BLOCKS: 6-10 MEV |
| G6009 | RADIATION TREATMENT DELIVERY, 2 SEPARATE TREATMENT AREAS, 3 OR MORE PORTS ON A SINGLE TREATMENT AREA, USE OF MULTIPLE BLOCKS: 11-19 MEV |
| G6010 | RADIATION TREATMENT DELIVERY, 2 SEPARATE TREATMENT AREAS, 3 OR MORE PORTS ON A SINGLE TREATMENT AREA, USE OF MULTIPLE BLOCKS: 20 MEV OR GREATER |
| G6011 | RADIATION TREATMENT DELIVERY,3 OR MORE SEPARATE TREATMENT AREAS, CUSTOM BLOCKING, TANGENTIAL PORTS, WEDGES, ROTATIONAL BEAM, COMPENSATORS, ELECTRON BEAM; UP TO 5 MEV |
| G6012 | RADIATION TREATMENT DELIVERY,3 OR MORE SEPARATE TREATMENT AREAS, CUSTOM BLOCKING, TANGENTIAL PORTS, WEDGES, ROTATIONAL BEAM, COMPENSATORS, ELECTRON BEAM; 6-10 MEV |
| G6013 | RADIATION TREATMENT DELIVERY,3 OR MORE SEPARATE TREATMENT AREAS, CUSTOM BLOCKING, TANGENTIAL PORTS, WEDGES, ROTATIONAL BEAM, COMPENSATORS, ELECTRON BEAM; 11-19 MEV |
| G6014 | RADIATION TREATMENT DELIVERY,3 OR MORE SEPARATE TREATMENT AREAS, CUSTOM BLOCKING, TANGENTIAL PORTS, WEDGES, ROTATIONAL BEAM, COMPENSATORS, ELECTRON BEAM; 20 MEV OR GREATER |
| G6015 | INTENSITY MODULATED TREATMENT DELIVERY, SINGLE OR MULTIPLE FIELDS/ARCS,VIA NARROW SPATIALLY AND TEMPORALLY MODULATED BEAMS, BINARY, DYNAMIC MLC, PER TREATMENT SESSION |
| G6016 | COMPENSATOR-BASED BEAM MODULATION TREATMENT DELIVERY OF INVERSE PLANNED TREATMENT USING 3 OR MORE HIGH RESOLUTION (MILLED OR CAST) COMPENSATOR, CONVERGENT BEAM MODULATED FIELDS, PER TREATMENT SESSION |
| G6017 | INTRA-FRACTION LOCALIZATION AND TRACKING OF TARGET OR PATIENT MOTION DURING DELIVERY OF RADIATION THERAPY (EG,3D POSITIONAL TRACKING, GATING, 3D SURFACE TRACKING), EACH FRACTION OF TREATMENT |
| G8379 | DOCUMENTATION OF RADIATION THERAPY RECOMMENDED WITHIN 12 MONTHS OF FIRST OFFICE VISIT |
| G9895 | DOCUMENTATION OF MEDICAL REASON(S) FOR NOT PRESCRIBING/ADMINISTERING ANDROGEN DEPRIVATION THERAPY IN COMBINATION WITH EXTERNAL BEAM RADIOTHERAPY TO THE PROSTATE (E.G., SALVAGE THERAPY) |
| G9896 | DOCUMENTATION OF PATIENT REASON(S) FOR NOT PRESCRIBING/ADMINISTERING ANDROGEN DEPRIVATION THERAPY IN COMBINATION WITH EXTERNAL BEAM RADIOTHERAPY TO THE PROSTATE |
| G9897 | PATIENTS WHO WERE NOT PRESCRIBED/ADMINISTERED ANDROGEN DEPRIVATION THERAPY IN COMBINATION WITH EXTERNAL BEAM RADIOTHERAPY TO THE PROSTATE, REASON NOT GIVEN |
| Q0064 | REMOTE AFTERLOAD BRACHYTHERAPY, 1-4 SOURCE POSITIONS OR CATHETERS |
| Q0065 | REMOTE AFTERLOAD BRACHYTHERAPY, 5-8 SOURCE POSITIONS OR CATHETERS |
| Q0067 | REMOTE AFTERLOAD BRACHYTHERAPY, MORE THAN 12 SOURCE POSITIONS OR CATHETERS |
| Q0076 | REMOTE AFTERLOAD BRACHYTHERAPY, 9-12 SOURCE POSITIONS OR CATHETERS |
| Q3001 | Radioelements for brachytherapy, any type, each |
| S2270 | INSERTION OF VAGINAL CYLINDER FOR APPLICATION OF RADIATION SOURCE OR CLINICAL BRACHYTHERAPY |
| S8049 | INTRAOPERATIVE RADIATION THERAPY (SINGLE ADMINISTRATION) |
| Z0903 | UNLIST RADIATION THERAPY |
